# Polymorphism and selection of *rpoS *in pathogenic *Escherichia coli*

**DOI:** 10.1186/1471-2180-9-118

**Published:** 2009-06-03

**Authors:** Tao Dong, Sarah M Chiang, Charlie Joyce, Rosemary Yu, Herb E Schellhorn

**Affiliations:** 1 Department of Biology, Life Sciences Building, Rm. 433, McMaster University, 1280 Main Street West, Hamilton, ON, L8S 4K1, Canada

## Abstract

**Background:**

Though RpoS is important for survival of pathogenic *Escherichia coli *in natural environments, polymorphism in the *rpoS *gene is common. However, the causes of this polymorphism and consequential physiological effects on gene expression in pathogenic strains are not fully understood.

**Results:**

In this study, we found that growth on non-preferred carbon sources can efficiently select for loss of RpoS in seven of ten representative verocytotoxin-producing *E. coli *(VTEC) strains. Mutants (Suc^++^) forming large colonies on succinate were isolated at a frequency of 10^-8 ^mutants per cell plated. Strain O157:H7 EDL933 yielded mainly mutants (about 90%) that were impaired in catalase expression, suggesting the loss of RpoS function. As expected, inactivating mutations in *rpoS *sequence were identified in these mutants. Expression of two pathogenicity-related phenotypes, cell adherence and RDAR (red dry and rough) morphotype, were also attenuated, indicating positive control by RpoS. For the other Suc^++ ^mutants (10%) that were catalase positive, no mutation in *rpoS *was detected.

**Conclusion:**

The selection for loss of RpoS on poor carbon sources is also operant in most pathogenic strains, and thus is likely responsible for the occurrence of *rpoS *polymorphisms among *E. coli *isolates.

## Background

Adaptation is important for survival of bacteria in various natural environments, but the underlying mechanisms are not fully understood. Bacteria are often present in large communities (e.g., biofilm [1]) in nature, and adaptation can occur at population levels. An important adaptive strategy is the generation of variants to maximize bacteria fitness at the population level in response to fluctuating environments [2,3]. These variants may result from spontaneous mutations selected within a population or from non-genetic changes. For example, to evade host immune system, some pathogens can alter surface antigen structure [4], termed phase variation [4,5], through revertible high frequency mutation of genes encoding surface proteins [2,5]. Bacteria also exhibit cell-to-cell variation in gene expression, termed individuality [2], even in an isogenic population. For example, under suboptimal induction conditions, the *lac *operon in *Escherichia coli *exhibits two distinct expression states, either fully induced or non-induced, but not an intermediate [6]. Gene expression noise due to stochastic events also results in phenotypic variation within isogenic *E. coli *populations [2,7]. Both genetic selection and individuality are likely important for bacterial adaptation in natural environments [2].

An important adaptation regulator is the alternative sigma factor RpoS widely found in *E. coli *and many other proteobacteria [8,9]. RpoS controls a large regulon [10-14] and plays a critical role in survival against stresses, such as prolonged starvation [15], low pH [16], thermal stress [17], near-UV exposure [18] and oxidative stress [18]. Despite the importance of RpoS, many attenuating mutations in the *rpoS *gene have been identified in both laboratory and natural *E. coli *strains. For example, some K12 strains possess an amber mutation (TAG) at codon 33 [19], while others have Glu (GAG), Tyr (TAT), or Gln (GAG) at the same position [19,20]. GAG is commonly found in natural non-K12 *E. coli *isolates [19,20]. Mutations in *rpoS *have also been identified in Shiga-like toxin-producing *E. coli *strains [21].

Polymorphism of *rpoS *appears to be paradoxical to the central role that RpoS plays in survival. Mutants of *rpoS *can be selected under nutrient limitation and exhibit enhanced metabolic potential [22], suggesting a regulatory trade-off for fitness between stress resistance and nutrient scavenging [22]. Growth on weak acids, including succinate [23] and acetate [24], strongly selects for mutations in *rpoS *in laboratory *E. coli *strains [23]. Considering that the weak acid (e.g., acetate) concentration is relatively high in human colon (80 mM) where *E. coli *colonize [25,26], *E. coli *may face a similar selective pressure within the host environment. Selection for loss and gain of RpoS function may be an important adaptive mechanism, like phase variation, to ensure that *E. coli *can survive in complex natural environments.

However, whether this selection is responsible for the observed *rpoS *polymorphism in natural *E. coli *isolates remains unclear, primarily because most studies have been done with laboratory *E. coli *K12 strains. The genomes of *E. coli *isolates differ substantially and constitute a pangenome consisting of 13,000 genes, of which 2,200 genes are conserved among all isolates [27]. Since RpoS mostly controls expression of genes encoding non-essential functions [8,9,12,13], RpoS likely plays a considerable role in the expression of non-conserved genes in the pangenome. Given that *E. coli *K12 strains only possess about 1/3 of all genes found in the pangenome of *E. coli *[27], it is possible that *rpoS *selection is limited to laboratory strains. Interestingly, selection for *rpoS *could not be observed in a natural *E. coli *isolate ECOR10 under nutrient limitation (see Fig 5 in [22]).

In this study, we wished to address three outstanding questions. First, can *rpoS *mutants be selected in clinical strains isolated from natural environments? Of particular interest is whether this selection occurs in pathogenic strains, which may have important medical relevance because of the potential role of RpoS in bacterial pathogenesis. Second, are there other factors involved in the selection for enhanced metabolic abilities in natural strains? Finally, is there any evidence that this selection occurs in natural environments? To address these questions, we employed a succinate selection strategy as a tool [23] and examined the selection using a group of ten representative verocytotoxin-producing *E. coli *(VTEC) strains from all five identified seropathotypes as our model strains. VTEC strains, including the O157:H7 serotype, are responsible for most *E. coli *foodborne outbreaks and can cause severe diseases, including diarrhea, hemorrhagic colitis and the hemolytic uremic syndrome [28]. Our results show that the selection for loss of RpoS is operant in most pathogenic *E. coli *strains. Virulence traits including RDAR morphotype and cell adherence were attenuated as a result of *rpoS *mutations. In addition, although *rpoS *mutants constituted most of the metabolic enhanced mutants, there was a small fraction of mutants that had intact RpoS function, indicating that other factors can also increase metabolic potential under conditions examined. Interestingly, three of ten tested VTEC strains grew well on succinate, and no growth-enhanced mutants could be selected. One of these three strains possessed a null *rpoS *mutation. This indicates that an adaptation to poor carbon source may have occurred in natural *E. coli *populations.

## Results

### Polymorphisms of *rpoS *in wild type VTEC strains

The ten representative VTEC strains examined in this study (Table 1) belong to five seropathotypes that have been categorized on the basis of virulence and outbreak frequency [29]. To test whether selection for loss of RpoS function can occur in these isolates, we first examined the *rpoS *sequences of these strains. Many nucleotide base substitutions were found in *rpoS *(Table 2). However, these substitutions did not result in changes in protein sequence, except for a single transversion (G to T) in strain N99-4390 which formed a premature stop codon, resulting in a loss of 86 amino acids at the C-terminal end of RpoS. As expression of catalase HPII encoded by *katE *is highly RpoS-dependent [30,31], catalase production in all strains could be used to assess RpoS activity using plate catalase assays. Only N99-4390 exhibited a low catalase activity, consistent with the expected effect of the identified mutation in this strain. All tested VTEC strains were found to have a GAG at codon 33, in contrast to CAG in the laboratory K12 strain MG1655 (Table 2).

**Table 1:** Suc^++ ^mutants selected from VTEC strains with attenuated or intact RpoS functions.

Sero-pathotype	Serotype	Strain	Source	Host	Number of mutants		Ratio of *rpoS*/Suc^++^
						
					Suc^++^	*rpoS*	
A	O157:H7	EDL933	J. Kaper	Human	12	11	0.92
B	O121:H19	CL106	LFZ	Human	12	10	0.83
	O111:NM	R82F2	LFZ	Human	N/A		N/A
C	O5:NM	N00-4067	BCCDC, NLEP	Human	12	12	1.00
	O113:H21	CL3	LFZ	Human	N/A		N/A
	O121:NM	N99-4390	BCCDC, NLEP	Human	N/A		N/A
D	O103:H25	N00-4859	BCCDC, NLEP	Human	12	12	1.00
	O172:NM	EC6-484	LFZ	Bovine	12	8	0.67
E	O84:NM	EC2-044	LFZ	Bovine	12	12	1.00
	O98:H25	EC3-377	LFZ	Bovine	12	12	1.00

Twelve Suc^++ ^mutants from each strain were tested for catalase activity using a plate catalase assay. Mutants impaired in catalase were considered as putative *rpoS *mutants. Detailed VTEC strain information is described elsewhere [29].

**Table 2:** Polymorphic codons in *rpoS *among VTEC strains.

Codon	33	54	119	129	154	181	191	243	273	317
	Glu	Val	Leu	Arg	Ile	Thr	His	Glu	Val	Leu
Consensus	GAG	GTG	CTT	CGC	ATT	ACC	CAT	GAG	GTG	CTG
MG1655	**C . .**	**. . .**	**. . .**	**. . .**	**. . .**	**. . .**	**. . .**	**. . .**	**. . .**	**. . .**
EDL933	**. . .**	**. . .**	**. . .**	**. . **T	**. . .**	**. . **A	**. . .**	**. . .**	**. . **A	**. . .**
CL106	**. . .**	**. . .**	**. . .**	**. . .**	**. . .**	**. . .**	**. . .**	**. . .**	**. . .**	**. . .**
R82F2	**. . .**	**. . **A	**. . .**	**. . .**	**. . .**	**. . .**	**. . .**	**. . .**	**. . .**	**. . .**
N00-4067	**. . .**	**. . .**	**. . .**	**. . .**	**. . .**	**. . .**	**. . .**	**. . .**	**. . .**	**. . **A
CL3	**. . .**	**. . .**	**. . .**	**. . .**	**. . .**	**. . .**	**. . .**	**. . .**	**. . .**	**. . .**
N99-4390	**. . .**	**. . .**	**. . **G	**. . .**	**. . **C	**. . .**	**. . **C	**T . .**		
N00-4859	**. . .**	**. . .**	**. . .**	**. . .**	**. . .**	**. . .**	**. . .**	**. . .**	**. . .**	**. . .**
EC6-484	**. . .**	**. . .**	**. . .**	**. . .**	**. . .**	**. . .**	**. . .**	**. . .**	**. . .**	**. . **A
EC2-044	**. . .**	**. . .**	**. . .**	**. . .**	**. . .**	**. . .**	**. . .**	**. . .**	**. . .**	**. . .**
EC3-377	**. . .**	**. . .**	**. . .**	**. . .**	**. . .**	**. . .**	**. . .**	**. . .**	**. . .**	**. . .**

The *rpoS *gene in *E. coli *K-12 MG1655 strain was used as the reference for comparison. The G-C transition at codon 33 in MG1655 results in a conversion of glutamate to glutamine, while the G-T transversion in N99-4390 at codon 243 forms a stop codon resulting in a truncated RpoS protein. The other polymorphic sites are synonymous mutations.

### Selection of Suc^++ ^mutants

Our primary goal was to determine if loss of RpoS in VTEC strains can be selected by growing cells on non-preferred carbon sources. Mutants forming large colonies (Suc^++^) were readily isolated from seven of ten tested strains at a frequency of 10^-8 ^per cell plated on succinate media, consistent with the frequencies obtained for laboratory strains [23]. Interestingly, strains CL3, R82F2 and N99-4390 grew uniformly well on succinate plates, much better than the other wild type strains, thus no Suc^++ ^mutants were obtained. Similar results were obtained by growing cells on fumarate, another TCA cycle intermediate (data not shown), indicating that this selection is not limited to succinate alone.

**Figure 1:**
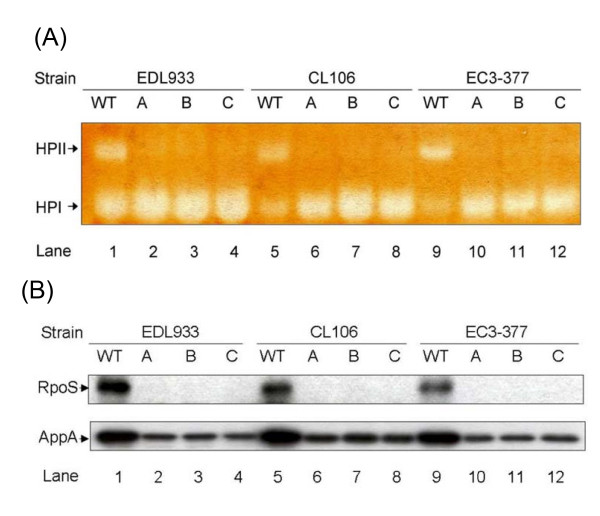
**Catalase activity and RpoS expression in representative Suc^++ ^mutants of VTEC strains EDL933, CL106 and EC3-377**. (A) Samples were separated by native PAGE and stained for catalase activity. Catalase HPI (KatG) and HPII (KatE) are indicated. (B) Expression of RpoS and RpoS-regulated AppA by Western analysis. Mutations in *rpoS *were identified in these tested Suc^++ ^mutants by sequencing, and sequences are provided in Supplemental material Figure S1 and Figure S2. To confirm equal protein loading, identical gels were run in parallel and stained by Coomassie Blue R-250 [14,71].

A group of 12 independent representative Suc^++ ^mutants were selected from each strain to test their RpoS status using catalase plate assays [23]. Most of the Suc^++ ^mutants (depending on parental strain background) were impaired in catalase production (Table 1). In *E. coli*, there are two catalases, HPI (KatG) and HPII (KatE), but only catalase HPII (KatE) is highly RpoS-dependent [23]. To confirm the plate assay results and to differentiate between the expression of KatE and KatG, we tested the catalase activity in the isolated catalase-negative Suc^++ ^mutants from three representative VTEC strains EDL933, CL106, and EC3-377 using native-PAGE gels. As expected, all Suc^++ ^mutants exhibited substantially reduced HPII catalase activity (Figure 1A). The higher expression of HPI in Suc^++ ^mutants (Figure 1A) is not entirely unexpected. Low levels of HPII may lead to higher accumulation of intracellular hydrogen peroxide which can activate OxyR, the main regulator of HPI [32].

The enhanced growth of Suc^++ ^mutants was assessed in liquid media by comparing the growth of wild type EDL933 and the derived mutants. There was no difference between growth of mutants and wild type cultures on glucose. However, growth of wild type strains on succinate was much lower compared with that of mutant strains, with a 10-fold longer generation time (Table 3). In addition, the Suc^++ ^mutants grew similarly to an *rpoS*-null deletion mutant on succinate and glucose (Table 3).

**Table 3:** Growth of EDL933 and isogenic mutants in M9 minimal media with glucose, succinate, fumarate or malate as the sole carbon source.

Substrate	Generation time (min)		
	
	WT	*rpoS*	Suc^++^
Glucose	94 ± 8	102 ± 28	106 ± 8
Succinate	1,443 ± 250	93 ± 10	116 ± 14
Fumarate	2,780 ± 422	135 ± 12	139 ± 6
Malate	2,107 ± 731	1,443 ± 31	1,147 ± 16

M9 minimal media with glucose (0.4%), succinate (1%), fumarate (1%), or malate (1%) were prepared as described in Methods. Cells were grown in LB to an OD_600 _of 0.6, washed with 1× M9 salts at 4°C, and inoculated into fresh minimal media at a starting OD600 nm of 0.05. Cultures were incubated at 37°C and sampled every hour. This experiment was performed in triplicate.

### Characterization of *rpoS *mutations in Suc^++ ^mutants

To determine if the loss of RpoS function in Suc^++ ^mutants resulted from acquired mutations in *rpoS*, the *rpoS *region of VTEC Suc^++ ^mutants exhibiting catalase deficiency was amplified and sequenced in both directions. Inactivating mutations, predicted to result in premature termination of RpoS, were identified in the *rpoS *gene in all the Suc^++ ^catalase deficient mutants (see Additional files 1 and 2). These acquired mutations included transitions, transversions, deletions and duplications (see Additional files 1 and 2). To ensure that enhanced growth on succinate was attributable to acquisition of *rpoS *mutations (rather than to secondary mutations), selected Suc^++ ^mutants carrying *rpoS *null mutations were complemented with a plasmid-borne functional *rpoS *[33]. As expected, the growth of transformed cells on succinate was much slower than that of the Suc^++ ^parental strains, confirming that acquired mutations in *rpoS *are responsible for the enhanced growth of Suc^++ ^mutants (data not shown). To examine the effect of mutation on RpoS levels, Western analysis using polyclonal antisera to RpoS was performed. In the selected representative Suc^++ ^mutants (see Additional file 2), RpoS protein was absent (Figure 1B). In addition, the expression of AppA, a RpoS-dependent protein which has both acid phosphatase and phytase activities [34,35], was substantially decreased in Suc^++ ^mutants to about 25% of the expression level in isogenic wild type strains (Figure 1B).

### Growth of VTEC strains and derivative Suc^++ ^mutants under aerobic and anaerobic conditions

**Figure 2:**
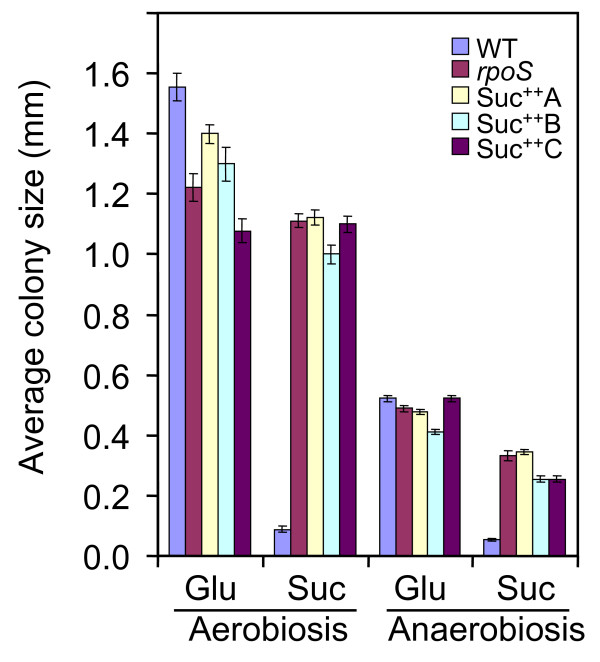
**Growth of EDL933 and derivative Suc^++ ^mutants on M9 glucose (Glu) and succinate (Suc) media**. Colony size (diameter) was determined under a light microscope at 40× magnification.

Effective utilization of succinate as a carbon source depends on the availability of an external electron receptor such as oxygen. However, in the human intestine, low oxygen tension permits *E. coli *to grow by fermentation or respiration using an alternative electron acceptor. As nitrate is readily available in the human intestine (14 μmol/kg [36]) and can be readily utilized by intestinal bacterial flora including *E. coli *[37,38] we examined succinate selection using this alternate electron receptor. Interestingly, host nitrate synthesis can be stimulated in response to infections caused by gastroenteric pathogens [38]. To test if selection for loss of RpoS can occur under low oxygen conditions, cultures were grown in anaerobic jars (see Methods). We first compared the anaerobic growth of wild type and aerobically-selected Suc^++ ^mutants on glucose and succinate plates. Wild type EDL933 grew as well as an isogenic *rpoS *knockout mutant and derivative Suc^++ ^mutants on glucose, while the *rpoS *and Suc^++ ^mutants grew much better than wild type on succinate under both aerobic and anaerobic conditions (Figure 2). The growth of Suc^++ ^mutants was similar to that of the control *rpoS *null mutant under all conditions tested.

All VTEC strains were then tested for selection on succinate under anaerobic conditions. As under aerobic conditions, Suc^++ ^mutants could be selected from all tested strains, except for CL3, R82F2 and N99-4390. Most (87%) of the Suc^++ ^had reduced catalase activity. We sequenced the *rpoS *region of 15 Suc^++ ^mutants isolated from EDL933 and found mutations in *rpoS*, resulting in impaired RpoS function, in 13 mutants while the *rpoS *gene in the other two Suc^++ ^mutants remained unchanged (data not shown).

### Expression of virulence-related traits, RDAR and cell adherence

**Figure 3:**
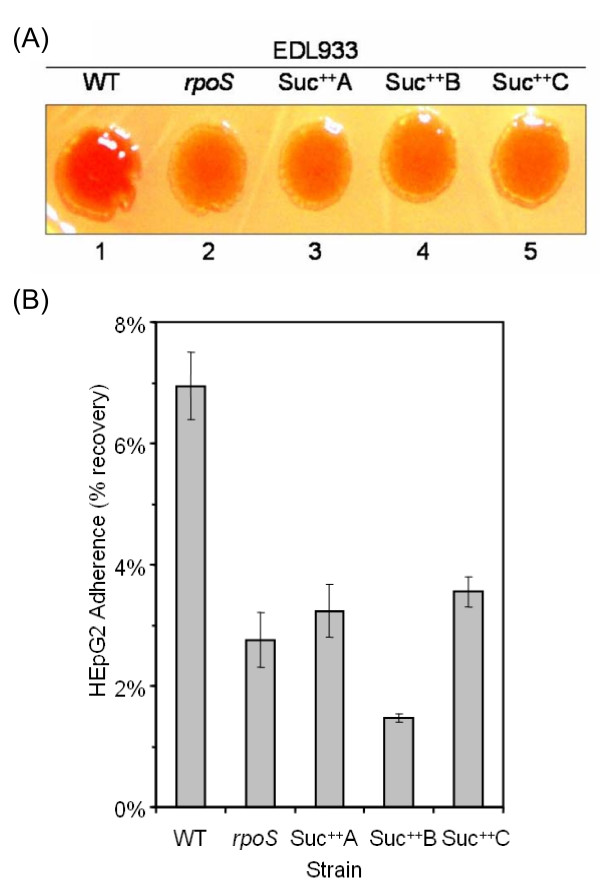
**Virulence-related traits, RDAR and cell adherence**. (A) Development of RDAR morphotype is impaired in Suc^++ ^mutants. Cells were replica-plated on CR (Congo Red) plates and incubated at 25°C for 48 h. (B) Cell adherence to epithelial cells. The adherence was expressed as the percentage of cells surviving the washing process. *rpoS *designates the constructed *rpoS *null-deletion mutant.

Mutations in *rpoS *may affect virulence factor expression in pathogenic strains [39,40]. To test this, we examined two virulence-related traits, the RDAR morphotype and cell adherence. Extracellular components, such as curli fimbriae and cellulose, are correlated with biofilm formation and virulence in *Salmonella sp*. and *E. coli *strains [41-43]. The expression of curli and cellulose can be visualized by staining with Congo Red dye to produce a red, dry and rough morphotype (RDAR) [43,44]. Biosynthesis of both curli and cellulose is positively regulated by RpoS through a transcriptional regulator CsgD in *E. coli *K12 [45,46]. However, to our knowledge, the role of RpoS in expression of RDAR has not been previously tested in pathogenic *E. coli *isolates. Wild type EDL933 exhibited a more pronounced RDAR morphotype than an isogenic *rpoS *null deletion mutant and Suc^++ ^mutants (Figure 3A), suggesting that RpoS is important for RDAR development. Similar results were also obtained for other VTEC strains (data not shown). Cell adherence assays were performed using human liver epithelial cell HepG2. The adherence of wild type EDL933 to HepG2 cells in tissue culture was two-fold higher than that of *rpoS *and Suc^++ ^mutants (P < 0.05) (Figure 3B), indicating that Suc^++ ^mutants are impaired in cell adherence due to loss of RpoS function. This is consistent with previous results that over-expression of RpoS stimulates cell adherence [47].

### Suc^++ ^mutants with an intact RpoS function (*rpoS*^+^)

During the screening for the Suc^++ ^phenotype, we found that a small proportion of Suc^++ ^mutants from strains EDL933 (8%), CL106 (16%), and EC6-484 (33%) were catalase-positive, a presumptive indication that RpoS was functional. To confirm this, we sequenced the *rpoS *region of five such Suc^++ ^mutants (three aerobically isolated and the other two anaerobically isolated) of strain EDL933. As expected, there was no mutation in the *rpoS *gene in these mutant strains. However, these grew much better than wild type when grown on succinate (generation time: 240 ± 31 min) and fumarate (generation time: 306 ± 33 min) (Table 3). These data suggest that non-*rpoS *mutations are a minor component in the poor carbon selection process.

### Effect of the *rpoS *mutation on metabolism by Phenotype Microarray analysis

RpoS is known to negatively control many genes involved in metabolism [10,12,48], and therefore, mutations in *rpoS *are likely to exert pleiotropic effects on metabolism. To test this, we compared wild type MG1655 and its derivative *rpoS *deletion mutants [12] using Phenotype Microarray analysis (Biolog, Hayward, CA). The *rpoS *mutants exhibited better respiration on 8 carbon sources and 92 nitrogen sources but less respiration on four carbon sources and one nitrogen source (Table 4). The substantial impact of *rpoS *mutations on nutrient utilization suggest that the beneficial effect of loss of RpoS in one selection condition may be extended to other conditions as well.

**Table 4:** Phenotypic Microarray (PM) analyses of growth changes resulted from *rpoS *mutations.

Carbon source	PM-value	Nitrogen source	PM-value
β-Methyl-D-Glucuronic Acid	102	Gly-Phe-Phe	157
L-Galactonic Acid-g-Lactone	98	Guanosine	137
L-Threonine	92	Nitrite	133
L-Alaninamide	70	D-Valine	125
L-Glutamine	67	Phe-b-Ala	124
L-Proline	66	L-Tyrosine	124
D-Trehalose	64	Tyr-Phe	120
D-Saccharic Acid	50	Phe-Phe	119
Propionic Acid	-51	Tyr-lle	118
Glycyl-L-Proline	-69	L-Glutamic Acid	113
α-Keto-Butyric Acid	-86	Ser-Gln	-67
α-Hydroxy-Butyric Acid	-110		

PM-value shows the growth difference between WT and *rpoS *mutants on these nutrients as carbon or nitrogen sources. Positive values show phenotypes gained in *rpoS *mutants while negative values show phenotypes lost because of *rpoS *mutations. In total, *rpoS *mutants grew better on 92 nitrogen sources tested, and the top 10 are listed.

### Enhanced growth of Suc^++ ^(*rpoS*^+ ^and *rpoS*^-^) mutants is not limited to the TCA cycle intermediates

**Figure 4:**
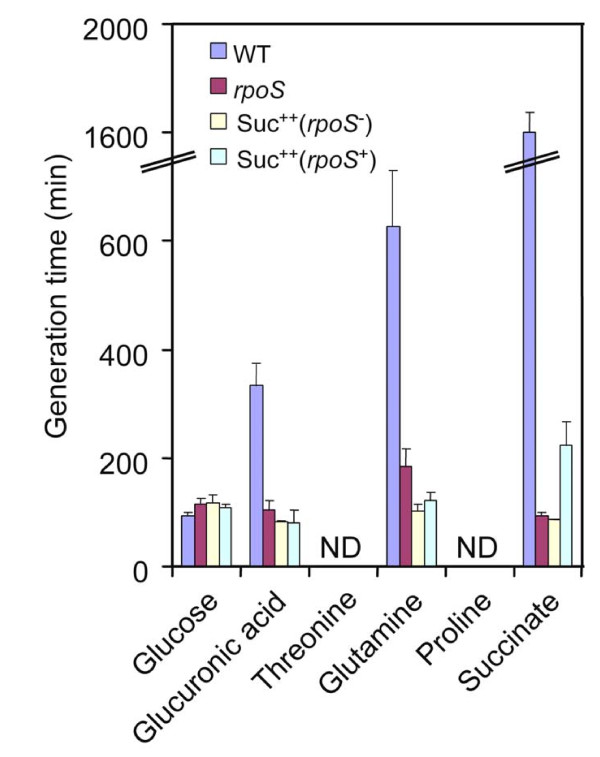
**Growth of EDL933 and derivative mutants on different carbon sources**. "ND": not detected. Cells were grown in LB media to OD_600 _0.6, washed and inoculated to fresh media to a starting OD_600_of 0.05. Cultures were then grown at 37°C with vigorous shaking (200 rpm) and sampled every hour for 10 hours to monitor growth. D-glucuronic acid, threonine, glutamine or proline were added to M9 minimal media as the sole carbon source to a final concentration of 20 mM.

To extend the phenotype screening results to pathogenic *E. coli*, we tested the growth of EDL933 and derivative *rpoS *and Suc^++ ^(*rpoS*^+ ^and *rpoS*^-^) mutants on selected carbon sources (20 mM each) that best supported differential respiration of *rpoS *mutants relative to wild type (Figure 4). Glucose and succinate were also tested as controls for comparison. As expected, compared with wild type, the *rpoS *and Suc^++ ^mutants grew similarly on glucose but much better on succinate. Among the Biolog compounds tested, the *rpoS *and Suc^++ ^mutants, including the Suc^++^(*rpoS*^+^) mutants, grew better than wild type on D-glucuronic acid or glutamine as the sole carbon source. However, none of these strains could grow on threonine or proline as the sole carbon source, which is likely due to differences in strain background and experimental conditions. The enhanced growth of mutants on D-glucuronic acid and glutamine confirmed that mutations selected on succinate have pleiotropic effects on utilization of other nutrient sources.

## Discussion

Understanding how pathogens adapt and mutate in response to growth environments is critical in deciphering many of the unknowns regarding pathogenesis, such as the emergence of new pathogens, the increased resistance to antibiotics, and the long-term persistence in host environment. In this study, we report that a metabolic selection mechanism for loss of RpoS, a central stress and adaptation regulator, in representative verocytotoxin-producing *E. coli *strains, may be responsible for the occurrence of *rpoS *mutations among pathogenic *E. coli *isolates. In surveying the *rpoS *gene among *E. coli *isolates, we found many mutations in *rpoS*, some of which result in loss of RpoS function. Among the VTEC strains tested, most grow poorly on succinate (like laboratory K12 strains) but some strains grow well. Those that grow poorly all have intact *rpoS*. In contrast, strains that grow well on succinate can be distinguished into two groups, one with intact *rpoS *and the other with truncated *rpoS*. The difference in utilization of succinate and *rpoS *status of these natural isolates is likely the result of certain selection that has occurred in natural environments. By testing growth-enhanced mutants (Suc^++^) selected from strains with intact *rpoS *on succinate, we identified two groups of mutants, one with impaired RpoS while the other with functional RpoS, a finding that is in agreement with the two parallel groups found in natural VTEC isolates. This correlation provides support that metabolic selection is a natural process relevant to pathogenic strains.

Most of the selected Suc^++ ^mutants had lost RpoS function, confirmed by both DNA sequencing and Western analyses. The positive selection pressure for *rpoS *mutations may result from the known negative effect of RpoS on a large group of genes including those in the TCA cycle [10,12,48,49]. In *E. coli*, the number of sigma factors greatly exceeds the number of RNA core polymerase, and thus there is a strong competition among sigma factors for binding to the core polymerase [50]. Genes involved in the TCA cycle are primarily transcribed by RpoD, the vegetative sigma factor [50]. The absence of RpoS, caused by *rpoS *mutation or low levels of expression, may thus result in an increase in RpoD-associated RNA polymerase, thereby leading to enhanced expression of the TCA cycle genes [12,51,52].

Mutations in *rpoS *result in substantial phenotypic modification. A previous study using similar Biolog screening technology has shown that the mutation of *rpoS *stimulates metabolism of about 20 carbon compounds in some *E. coli *strains but only has a minor effect in MG1655 [22]. By comparing respiration rates instead of final OD employed in the previous study, we extended previous results and found that the respiration of the *rpoS *deletion mutant [12] increased in over 100 new compounds compared with wild type MG1655. Thus, we suggest that RpoS, known as a master stress regulator, can be also envisioned as a central metabolism repressor, whose inactivation results in enhanced nutrient utilization abilities. RpoS, therefore, is a critical control in cellular fitness, which can be defined as better survival or growth depending on environmental conditions. During stress conditions, activation of RpoS promotes survival by protecting cells from multiple stresses. During growth on poor carbon sources, however, mutating RpoS results in better growth by conferring cells enhanced metabolic abilities. In either case, cell fitness is effectively achieved through modulation of a single factor, RpoS.

What are the potential effects for loss of RpoS in pathogenic *E. coli*? On one hand, mutations in *rpoS *in Suc^++ ^mutants may attenuate RpoS-mediated stress resistance and virulence functions. Suc^++ ^mutants were deficient in RDAR morphotype development, an indicator for expression of extracellular components that are important for bacterial pathogenesis [41]. We also found that adherence to epithelial cells was impaired in *rpoS *and Suc^++ ^mutants, indicating a decrease in pathogenesis. On the other hand, because *rpoS *mutants can better utilize non-preferred carbon sources [23], *rpoS *mutations may help *E. coli *compete with other bacteria in the human intestine, a highly-competitive environment harboring at least 1,000 different species [53]. It has been reported that *rpoS *mutants outcompete wild type strains in colonizing mouse intestine [54]. Although mutations in *rpoS *may increase the sensitivity of *E. coli *cells to exogenous stresses (due to the loss of protective functions such as catalase), enhanced metabolism of less-preferred carbon sources may offset this deficiency and lead to, on the whole, selection for *rpoS *mutations even in a competitive environment [52]. This has led to the proposal by Ferenci and co-workers that the loss of RpoS may be viewed as an increase in metabolic fitness at the expense of a loss of protective functions [55]. A slightly different scenario may be operant in VTEC strains where loss of pathogenic functions, such as curli fimbriae, may occur during selection for enhanced metabolic fitness (this study), even in the host environment where *rpoS *mutants can be isolated [21]. It is also important to note that mutants of *rpoS *were isolated at a low frequency close to spontaneous mutation frequency (10^-8^), suggesting that naturally occurred *rpoS *mutants would constitute, at least initially, only a small fraction of *E. coli *population unless there is a prolonged strong selective condition (i.e., poor carbon source).

**Figure 5:**
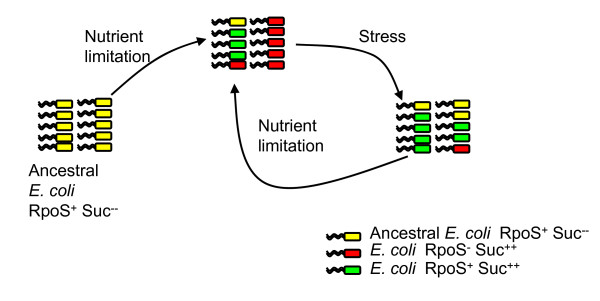
**Dynamic view of RpoS status and metabolic fitness in natural *E. coli *populations**. It is postulated that the ancestral *E. coli *strain possesses a functional *rpoS *allele (RpoS^+^) but cannot grow well on poor nutrient sources (Suc^--^). Upon exposure to nutrient limitation, mutants (Suc^++^) exhibiting enhanced metabolic activity can be selected and become dominant among the population. These mutants consist of two groups, RpoS^+ ^and RpoS^-^. Under stress conditions, however, the proportion of RpoS^- ^mutants decreases because of the loss of protection by RpoS-controlled functions, and the abundance of strains with functional RpoS increases. Because cells likely are constantly facing selection between nutrient limitation and stress in nature, the population of *E. coli *isolates is in a dynamic status in terms of RpoS function and metabolic fitness.

Although loss of RpoS appears to be the usual consequence of selection for metabolic fitness, clearly other mutation(s) can also occur and result in an enhanced growth phenotype (e.g., five of 30 EDL933-derived Suc^++ ^mutants characterized did not acquire mutations in *rpoS*). The occurrence of non-*rpoS *mutations may be strain-specific, since such mutations could not be selected from K12 strains [23] or from some of the tested VTEC strains in this study. The non-*rpoS *mutations may represent another adaptation strategy of *E. coli *in natural environments, in which metabolic fitness is achieved without the cost of RpoS-controlled stress resistance system (Figure 5). Of the ten tested wild type VTEC strains, three grew well on succinate, among which two strains (CL3 and R82F2) are RpoS^+ ^and one (N99-4390) is RpoS^-^. It is possible that both *rpoS *and non-*rpoS *mutations for enhanced growth could have occurred in nature among *E. coli *isolates. Given the importance of RpoS in cell survival, growth-enhanced mutations that retain RpoS functions may be better preserved among *E. coli *natural populations. Using representative natural commensal *E. coli *isolates from the ECOR collection [56], we recently found that seven of ten wild type ECOR strains can utilize succinate well; six of them were RpoS^+ ^and one was RpoS^- ^(Dong and Schellhorn, unpublished data).

## Conclusion

In summary, non-preferred carbon sources can select for *rpoS *mutations in pathogenic VTEC *E. coli *strains. The resultant Suc^++ ^mutants also exhibited growth advantages on succinate minimal media under anaerobic conditions with nitrate as a respiratory electron receptor. Suc^++ ^mutants harboring *rpoS *mutations were impaired in the development of RDAR morphotype and the ability of adherence to epithelial cells. Heterogeneity of *rpoS *as a result of the selection may thus contribute to differences in pathogenesis among pathogenic *E. coli *strains.

## Methods

### Bacterial strains, media, and growth conditions

Pathogenic strains examined in this study are listed in Table 1. Strains were routinely grown in Luria-Bertani (LB) broth aerobically at 37°C with shaking at 200 rpm. Cell growth was monitored spectrophotometrically at 600 nm. M9 minimal media was supplemented with glucose (0.4% wt/vol), succinate (1%), fumarate (1%) or malate (1%) as a sole carbon source [57]. Media was supplemented with ampicillin (100 μg/ml) and chloramphenicol (25 μg/ml) as indicated. All chemicals and media were supplied by Invitrogen, Fisher Scientific, or Sigma-Aldrich. The generation time was determined using exponential phase cultures (*g *= *t*/(3.3 (log *N*-log *N*_0_)); *g *= generation time; *t *= time of exponential growth; *N*_0 _= initial cell number; *N *= final cell number) [58].

### HepG2 cell growth

HepG2 cells were grown at 37°C in 5% CO_2 _in Dulbecco's modified Eagle's medium (DMEM) supplemented with 10% (v/v) heat-inactivated fetal bovine serum (FBS).

### Selection of Suc^++ ^mutants

Cultures were inoculated into LB broth from single colonies. After overnight incubation, cells were washed 3 times with M9 minimal salts to eliminate media carryover, plated on succinate minimal media (approximately 10^9 ^cells) and incubated at 37°C for 48 h. Several large colonies (Suc^++^) from each plate were picked and purified by serial streaking on succinate plates. The selection for Suc^++ ^mutants was performed in triplicate using independent colonies to ensure isolated mutants were not clones descended from single variants. Three independent mutants, selected from independently-grown cultures of each strain, were sequenced using *rpoS *flanking primers as described below.

### Amplification of the *rpoS *region and sequencing

The *rpoS *region of wild type strains and putative *rpoS *mutants that were catalase-deficient was amplified using primers FP1 (CAACAAGAAGTGAAGGCGGG) and RP1 (TGGCCTTTCTGACAGAT GCTTAC) by whole colony PCR. A single colony from each strain was resuspended into 30 μl ddH_2_O, heated at 95°C for 5 min, and 4 μl was used in a standard 20 μl PCR reaction. PCR products were purified by QIAquick Purification Kit (Qiagen, Inc.) and sequenced by MOBIX lab (McMaster University).

### Construction of EDL933 *rpoS *deletion mutant

A precise *rpoS *deletion mutant of EDL933 was constructed using the Red recombination system [59], and served as a negative control for the following experiments. The *rpoS *gene was replaced by homologous recombination with the chloramphenicol resistant gene *cat*, which was amplified using pKD3 plasmid (the template) and primers FP2 (CCTCGCTTGAGACTG GCCTTTCTGACAGATGCTTACGTGTAGGCTGGAGCTGCTTC) and RP2 (ATGTTC CGTCAAGGGATCACGGGTAGGAGCCACCTTCATATGAATATCCTCCTTAG). The *cat *gene was further removed from the chromosome by recombination with the FLP recombinase. The resultant mutant lost the entire *rpoS *ORF. The mutation was confirmed by PCR using primers flanking the deleted region.

### Catalase assay

Native polyacrylamide gel electrophoresis (PAGE) was performed to examine the catalase activity in selected Suc^++ ^mutants. Overnight cultures were harvested by centrifugation at 4,000 × g for 15 min at 4°C, and washed three times in potassium phosphate buffer (50 mM, pH 7.0). Cells were resuspended to OD_600 *nm *_= 15 in potassium phosphate buffer (50 mM, pH 7.0) and disrupted by sonication using a Heat Systems sonicator (Misonix, Inc., Farmingdale, New York). Cell debris was removed by centrifugation for 15 min at 12,000 × g at 4°C. Protein concentration was determined by the Bradford assay using bovine serum albumin as a standard [60]. Ten μg of each protein sample were loaded on a 10% native polyacrylamide gel and resolved at 160 V for 50 min. The gel was then stained with horseradish peroxidase and diaminobenzidine as described by Clare *et al*. [61]. Parallel gels were stained with Coomassie Blue R-250 to verify equal protein loading. Plate catalase assays were used to qualitatively test the Suc^++ ^mutants for loss of catalase activity by dropping 10 μl of 30% H_2_O_2 _on the plates, an indicator for *rpoS *status because catalase production is highly-RpoS dependent [30].

### Western blot analysis

Protein samples were prepared as described for catalase staining. Samples (10 μg) were boiled for 5 min, loaded on a 10% SDS-PAGE gel, and fractioned at 160 V for 50 min. Protein samples were then transferred from the gel onto a PVDF membrane by electrophoresis at 90 V for 1 h. The PVDF membrane was incubated with anti-RpoS (a gift from R. Hengge, Freie Universität Berlin) or anti-AppA sera (a gift from C.W. Forsberg, University of Guelph) and secondary antibody of goat anti-rabbit immunoglobulin (Bio-Rad). Signals were detected using enhanced chemiluminescence (Amersham Bioscience).

### Growth under aerobic and anaerobic conditions

Culture samples were collected after overnight incubation in LB media, and washed 3 times in M9 salts. To obtain isolated mutant colonies, serial dilutions were plated on M9 minimal media with either glucose (0.4%) or succinate (1%) as the sole carbon source, and incubated for 72 h at 37°C under aerobic or anaerobic conditions as indicated. Anaerobic conditions were maintained in Brewer anaerobic jars (Becton Dickinson) using the BBL GasPak anaerobic system as described previously [62]. Potassium nitrate (40 mM) was supplemented to all the media to provide an electron receptor for respiration under anaerobic conditions [62]. The diameter of individual colonies was determined at 40× magnification.

### Test of pathogenicity-related traits

#### (a) RDAR morphotype

To visualize RDAR (red, dry and rough) cell morphotype [44], a single colony of each strain was resuspended in non-salt LB media (1% tryptone and 0.5% yeast extract) in a 96-well microtiter plate, transferred to Congo Red (CR) plates (non-salt LB media with 1.5% agar, 40 μg/ml of Congo Red dye, and 20 μg/ml of Coomassie Blue R-250) by replica plating, and grown at 25°C for 48 h [44].

#### (b) Adherence assay

Quantitative adherence assays were performed as described by Torres and Kaper [63]. Wild type *E. coli *EDL933 and derivative *rpoS *and Suc^++ ^mutants were tested for adherence to human liver epithelial HepG2 cells. Confluent HepG2 cultures grown in DMEM were incubated with 10^8 ^CFU *E. coli *overnight grown cells for 6 h at 37°C in 5% CO_2_. Adhered *E. coli *cells were washed with PBS buffer, released by 0.1% Triton X-100 and enumerated by serial plating on LB media. The adherence is reported as the percentage of cells that remain adherent following the washing process. The statistical significance of differences between treatment groups was determined using an unpaired Student's *t*-test [64].

#### Phenotype Microarray analysis

To assess the effect of RpoS on metabolism, we compared wild type MG1655 *E. coli *strain and a derivative null-*rpoS *mutant [12] using a commercial high-throughput phenotype screening service, Phenotype Microarray (PM) analysis (Biolog, Hayward, CA), that permits evaluation of about 2,000 cellular phenotypes including utilization of carbon, nitrogen, phosphate and sensitivity to various stresses [65,66]. PM analysis assesses substrate-dependent changes in cell respiration using tetrazolium as an electron acceptor and has been widely used to test growth phenotypes [67-69].

#### Sequence alignment

The *rpoS *sequences of VTEC *E. coli *strains and isolated mutants were aligned by ClustalW [70] and graphically depicted using Vector NTI 10 (Invitrogen, Carlsbad, CA).

## Authors' contributions

TD performed most of the experiments and wrote the first draft. RY aided in sequencing the *rpoS *region of selected mutants. SMC, CJ, and HES helped in the design of several experiments and revision of the manuscript. HES is the principal investigator and supervised the project. All authors read and approved the final manuscript.

## Supplementary Material

Additional file 1 **Alignment of *rpoS *gene sequences of Suc^++ ^mutants with parental strains**. The alignment data show the location of mutations within the *rpoS *gene in the selected Suc^++ ^mutants in comparison with parental strains. Click here for file

Additional file 2 **Alignment of predicted RpoS protein sequences of Suc^++ ^mutants with parental strains**. The protein alignment data show the predicted mutant forms of RpoS resulting from the identified mutations in the *rpoS *gene of Suc^++ ^mutants. Click here for file
