# Supplementary material for: Polymorphism and selection of rpoS in pathogenic Escherichia coli
Source: BMC Microbiol. 2009 Jun 3;9:118. doi: 10.1186/1471-2180-9-118 (PMC2700278; doi:10.1186/1471-2180-9-118)
Supplement: Additional file 1 — Alignment of rpoS gene sequences of Suc++ mutants with parental strains. The alignment data show the location of mutations within the rpoS gene in the selected Suc++ mutants in comparison with parental strains. [file 1471-2180-9-118-S1.pdf]

Section 1

	(1)	1 10 20 30 40 50 60 70 86
EDL933	(1)	ATGAGTCAGAATACGCTGAAAGTTCATGATTTAAATGAAGATGCGGAATTTGATGAGAACGGAGTTGAGGTTTTTGACGAAAAGGC
EDL933A	(1)	ATGAGTCAGAATACGCTGAAAGTTCATGATTTAAATGAAGATGCGGAATTTGATGAGAACGGAGTTGAGGTTTTTGACGAAAAGGC
EDL933B	(1)	ATGAGTCAGAATACGCTGAAAGTTCATGATTTAAATGAAGATGCGGAATTTGATGAGAACGGAGTTGAGGTTTTTGACGAAAAGGC
EDL933C	(1)	ATGAGTCAGAATACGCTGAAAGTTCATGATTTAAATGAAGATGCGGAATTTGATGAGAACGGAGTTGAGGTTTTTGACGAAAAGGC
CL106	(1)	ATGAGTCAGAATACGCTGAAAGTTCATGATTTAAATGAAGATGCGGAATTTGATGAGAACGGAGTTGAGGTTTTTGACGAAAAGGC
CL106A	(1)	ATGAGTCAGAATACGCTGAAAGTTCATGATTTAAATGAAGATGCGGAATTTGATGAGAACGGAGTTGAGGTTTTTGACGAAAAGGC
CL106B	(1)	ATGAGTCAGAATACGCTGAAAGTTCATGATTTAAATGAAGATGCGGAATTTGATGAGAACGGAGTTGAGGTTTTTGACGAAAAGGC
CL106C	(1)	ATGAGTCAGAATACGCTGAAAGTTCATGATTTAAATGAAGATGCGGAATTTGATGAGAACGGAGTTGAGGTTTTTGACGAAAAGGC
EC3377	(1)	ATGAGTCAGAATACGCTGAAAGTTCATGATTTAAATGAAGATGCGGAATTTGATGAGAACGGAGTTGAGGTTTTTGACGAAAAGGC
EC3377A	(1)	ATGAGTCAGAATACGCTGAAAGTTCATGATTTAAATGAAGATGCGGAATTTGATGAGAACGGAGTTGAGGTTTTTGACGAAAAGGC
EC3377B	(1)	ATGAGTCAGAATACGCTGAAAGTTCATGATTTAAATGAAGATGCGGAATTTGATGAGAACGGAGTTGAGGTTTTTGACGAAAAGGC
EC3377C	(1)	ATGAGTCAGAATACGCTGAAAGTTCATGATTTAAATGAAGATGCGGAATTTGATGAGAACGGAGTTGAGGTTTTTGACGAAAAGGC
EC2044	(1)	ATGAGTCAGAATACGCTGAAAGTTCATGATTTAAATGAAGATGCGGAATTTGATGAGAACGGAGTTGAGGTTTTTGACGAAAAGGC
EC2044A	(1)	ATGAGTCAGAATACGCTGAAAGTTCATGATTTAAATGAAGATGCGGAATTTGATGAGAACGGAGTTGAGGTTTT-GACGAAAAGGC
EC2044B	(1)	ATGAGTCAGAATACGCTGAAAGTTCATGATTTAAATGAAGATGCGGAATTTGATGAGAACGGAGTTGAGGTTTTTGACGAAAAGGC
EC2044C	(1)	ATGAGTCAGAATACGCTGAAAGTTCATGATTTAAATGAAGATGCGGAATTTGATGAGAACGGAGTTGAGGTTTTTGACGAAAAGGC
EC6484	(1)	ATGAGTCAGAATACGCTGAAAGTTCATGATTTAAATGAAGATGCGGAATTTGATGAGAACGGAGTTGAGGTTTTTGACGAAAAGGC
EC6484A	(1)	ATGAGTCAGAATACGCTGAAAGTTCATGATTTAAATGAAGATGCGGAATTTGATGAGAACGGAGTTGAGGTTTTTGACGAAAAGGC
EC6484B	(1)	ATGAGTCAGAATACGCTGAAAGTTCATGATTTAAATGAAGATGCGGAATTTGATGAGAACGGAGTTGAGGTTTTTGACGAAAAGGC
EC6484C	(1)	ATGAGTCAGAATACGCTGAAAGTTCATGATTTAAATGAAGATGCGGAATTTGATGAGAACGGAGTTGAGGTTTTTGACGAAAAGGC
N004067	(1)	ATGAGTCAGAATACGCTGAAAGTTCATGATTTAAATGAAGATGCGGAATTTGATGAGAACGGAGTTGAGGTTTTTGACGAAAAGGC
N004067A	(1)	ATGAGTCAGAATACGCTGAAAGTTCATGATTTAAATGAAGATGCGGAATTTGATGAGAACGGAGTTGAGGTTTTTGACGAAAAGGC
N004067B	(1)	ATGAGTCAGAATACGCTGAAAGTTCATGATTTAAATGAAGATGCGGAATTTGATGAGAACGGAGTTGAGGTTTTTGACGAAAAGGC
N004067C	(1)	ATGAGTCAGAATACGCTGAAAGTTCATGATTTAAATGAAGATGCGGAATTTGATGAGAACGGAGTTGAGGTTTTTGACGAAAAGGC
N004859	(1)	ATGAGTCAGAATACGCTGAAAGTTCATGATTTAAATGAAGATGCGGAATTTGATGAGAACGGAGTTGAGGTTTTTGACGAAAAGGC
N004859A	(1)	ATGAGTCAGAATACGCTGAAAGTTCATGATTTAAATGAAGATGCGGAATTTGATGAGAACGGAGTTGAGGTTTTTGACGAAAAGGC
N004859B	(1)	ATGAGTCAGAATACGCTGAAAGTTCATGATTTAAATGAAGATGCGGAATTTGATGAGAACGGAGTTGAGGTTTTTGACGAAAAGGC
N004859C	(1)	ATGAGTCAGAATACGCTGAAAGTTCATGATTTAAATGAAGATGCGGAATTTGATGAGAACGGAGTTGAGGTTTTTGACGAAAAGGC

Section 2

```
               (87) 87          100       110       120       130       140       150       160       172
EDL933         (87) CTTAGTAGAAGAGGAACCCAGTGATAACGATTTGGCCGAAGAGGAACTGTTATCGCAGGGAGCCACACAGCGTGTGTTGGACGCGA
EDL933A        (87) CTTAGTAGAAGAGGAACCCAGTGATAACGATTTGGCCGAAGAGGAACTGTTATCGCAGGGAGCCACACAGCGTGTGTTGGACGCGA
EDL933B        (87) CTTAGTAGAAGAGGAACCCAGTGATAACGATTTGGCCGAAGAGGAACTGTTATCGCAGGGAGCCACACAGCGTGTGTTGGACGCGA
EDL933C        (87) CTTAGTAGAAGAGGAACCCAGTGATAACGATTTGGCCGAAGAGGAACTGTTATCGCAGGGAGCCACACAGCGTGTGTTGGACGCGA
CL106          (87) CTTAGTAGAAGAGGAACCCAGTGATAACGATTTGGCCGAAGAGGAACTGTTATCGCAGGGAGCCACACAGCGTGTGTTGGACGCGA
CL106A         (87) CTTAGTAGAAGAGGAACCCAGTGATAACGATTTGGCCGAAGAGGAACTGTTATCGCAGGGAGCCACACAGCGTGTGTTGGACGCGA
CL106B         (87) CTTAGTAGAAGAGGAACCCAGTGATAACGATTTGGCCGAAGAGGAACTGTTATCGCAGGGAGCCACACAGCGTGTGTTGGACGCGA
CL106C         (87) CTGA----------------------------------------------------------------------------------
EC3377         (87) CTTAGTAGAAGAGGAACCCAGTGATAACGATTTGGCCGAAGAGGAACTGTTATCGCAGGGAGCCACACAGCGTGTGTTGGACGCGA
EC3377A        (87) CTTAGTAGAAGAGGAACCCAGTGATAACGATTTGGCCGAAGAGGAACTGTTATCGCAGGGAGCCACACAGCGTGTGTTGGACGCGA
EC3377B        (87) CTTAGTAGAAGAGGAACCCAGTGATAACGATTTGGCCGAAGAGGAACTGTTATCGCAGGGAGCC-CACAGCGTGTGTTGGACGCGA
EC3377C        (87) CTTAGTAGAAGAGGAACCCAGTGATAACGATTTGGCCGAAGAGGAACTGTTATCGCAGGGAGCC-----GCGTGTGTTGGACGCGA
EC2044         (87) CTTAGTAGAAGAGGAACCCAGTGATAACGATTTGGCCGAAGAGGAACTGTTATCGCAGGGAGCCACACAGCGTGTGTTGGACGCGA
EC2044A        (86) CTTAG---------------------------------------------------------------------------------
EC2044B        (87) CTTAGTAGAAGAGGAACCCAGTGATAACGATTTGGCCGAAGAGGAACTGTTATCGCAGGGAGCCACACAGCGTGTGTTGGACGCGA
EC2044C        (87) CTTAGTAGAAGAGGAACCCAGTGATAACGATTTGGCCGAAGAGGAACTGTTATCGCAGGGAGCCACACAGCGTGTGTTGGACGCGA
EC6484         (87) CTTAGTAGAAGAGGAACCCAGTGATAACGATTTGGCCGAAGAGGAACTGTTATCGCAGGGAGCCACACAGCGTGTGTTGGACGCGA
EC6484A        (87) CTTAGTAGAAGAGGAACCCAGTGATAACGATTTGGCCGAAGAGGAACTGTTATCGCAGGGAGCCACACAGCGTGTGTTGGACGCGA
EC6484B        (87) CTTAGTAGAAGAGGAACCCAGTGATAACGATTTGGCCGAAGAGGAACTGTTATCGCAGGGAGCCACACAGCGTGTGTTGGACGCGA
EC6484C        (87) CTTAGTAGAAGAGGAACCCAGTGATAACGATTTGGCCGAAGAGGAACTGTTATCGCAGGGAGCCACACAGCGTGTGTTGGACGCGA
N004067        (87) CTTAGTAGAAGAGGAACCCAGTGATAACGATTTGGCCGAAGAGGAACTGTTATCGCAGGGAGCCACACAGCGTGTGTTGGACGCGA
N004067A       (87) CTTAGTAGAAGAGGAACCCAGTGATAACGATTTGGCCGAAGAGGAACTGTTATCGCAGGGAGCCACACAGCGTGTGTTGGACGCGA
N004067B       (87) CTTAGTAGAAGAGGAACCCAGTGATAACGATTTGGCCGAAGAGGAACTGTTATCGCAGGGAGCCACACAGCGTGTGTTGGACGCGA
N004067C       (87) CTTAGTAGAAGAGGAACCCAGTGATAACGATTTGGCCGAAGAGGAACTGTTATCGCAGGGAGCCACACAGCGTGTGTTGGACGCGA
N004859        (87) CTTAGTAGAAGAGGAACCCAGTGATAACGATTTGGCCGAAGAGGAACTGTTATCGCAGGGAGCCACACAGCGTGTGTTGGACGCGA
N004859A       (87) CTTAGTAGAAGAGGAACCCAGTGATAACGATTTGGCCGAAGAGGAACTGTTATCGCAGGGAGCCACACAGCGTGTGTTGGACGCGA
N004859B       (87) CTTAGTAGAAGAGGAACCCAGTGATAACGATTTGGCCGAAGAGGAACTGTTATCGCAGGGAGCCACACAGCGTGTGTTGGACGCGA
N004859C       (87) CTTAGTAGAAGAGGAACCCAGTGATAACGATTTGGCCGAAGAGGAACTGTTATCGCAGGGAGCCACACAGCGTGTGTTGGACGCGA
```

Section 3

```
            (173) 173    180       190       200       210       220       230       240                258
EDL933      (173) CTCAGCTTTACCTTGGTGAGATTGGTTATTCACCACTGTTAACGGCCGAAGAAGAAGTTTATTTTGCGCGTCGCGCACTGCGTGGA
EDL933A     (173) CTCAGCTTTACCTTGGTGAGATTGGTTATTCACCACTGTTAACGGCCGAAGAAGAAGTTTATTTTGCGCGTCGCGCACTGCGTGGA
EDL933B     (173) CTCAGCTTTACCT-----------------CACCACTGTTAACGGCCGAAGAAGAAGTTTATTTTGCGCGTCGCGCACTGCGTGGA
EDL933C     (173) CTCAGCTTTACCTTGGTGAGATTGGTTATTCACCACTGTTAACGGCCGAA-AAGAAGTTTATTTTGCGCGTCGCGCACTGCGTGGA
CL106       (173) CTCAGCTTTACCTTGGTGAGATTGGTTATTCACCACTGTTAACGGCCGAAGAAGAAGTTTATTTTGCGCGTCGCGCACTGCGTGGA
CL106A      (173) CTCAGCTTTAC-TTGGTGAGATTGGTTATTCACCACTGTTAA--------------------------------------------
CL106B      (173) CTCAGCTTTACCTTGGTGAGATTGGTTATTCACCACTGTTAACGGCCGAAGAAGAAGTTTATTTTGCGCGTCGCGCACTGCGTGGA
CL106C       (91) --------------------------------------------------------------------------------------
EC3377      (173) CTCAGCTTTACCTTGGTGAGATTGGTTATTCACCACTGTTAACGGCCGAAGAAGAAGTTTATTTTGCGCGTCGCGCACTGCGTGGA
EC3377A     (173) CTCAGCTTTACCTTGGTGAGATTGGTTATTCACCACTGTTAACGGCCGAAGAAGAAGTTTATTTTGCGCGTCGCGCACTGCGTGGA
EC3377B     (172) CTCAGCTTTACCTTGGTGAGATTGGTTATTCACCACTGTTAA--------------------------------------------
EC3377C     (168) CTCAGCTTTACCTTGGTGA-------------------------------------------------------------------
EC2044      (173) CTCAGCTTTACCTTGGTGAGATTGGTTATTCACCACTGTTAACGGCCGAAGAAGAAGTTTATTTTGCGCGTCGCGCACTGCGTGGA
EC2044A      (91) --------------------------------------------------------------------------------------
EC2044B     (173) CTCAGCTTTACCTTGGTGAGATTGGTTATTCACCACTGTTAACGGCCGAAGAAGAAGTTTATTTTGCGCGTCGCGCACTGCGTGGA
EC2044C     (173) CTCAGCTTTACCTTGGTGAGATTGGTTATTCACCACTGTTAACGGCCGAAGAAGAAGTTTATTTTGCGCGTCGCGCACTGCGTGGA
EC6484      (173) CTCAGCTTTACCTTGGTGAGATTGGTTATTCACCACTGTTAACGGCCGAAGAAGAAGTTTATTTTGCGCGTCGCGCACTGCGTGGA
EC6484A     (173) CTCAGCTTTACCTTGGTGAGATTGGTTATTCACCACTGTTAACGGCCGAAGAAGAAGTTTATTTTGCGCGTCGCGCACTGCGTGGA
EC6484B     (173) CTCAGCTTTACCTTGGTGAGATTGGTTATTCACCACTGTTAACGGCCGAAGAAGAAGTTTATTTTGCGCGTCGCGCACTGCGTGGA
EC6484C     (173) CTCAGCTTTACCTTGGTGAGATTGGTTATTCACCACTGTTAACGGCCGAAGAAGAAGTTTATTTTGCGCGTCGCGCACTGCGTGGA
N004067     (173) CTCAGCTTTACCTTGGTGAGATTGGTTATTCACCACTGTTAACGGCCGAAGAAGAAGTTTATTTTGCGCGTCGCGCACTGCGTGGA
N004067A    (173) CTCAGCTTTACCTTGGTGAGATTGGTTATTCACCACTGTTAACGGCCGAAGAAGAAGTTTATTTTGCGCGTCGCGCACTGCGTGGA
N004067B    (173) CTCAGCTTTACCTTGGTGAGATTGGTTATTCACCACTGTTAACGGCCGAAGAAGAAGTTTATTTTGCGCGTCGCGCACTGCGTGGA
N004067C    (173) CTCAGCTTTACCTTGGTGAGATTGGTTATTCACCACTGTTAACGGCCGAAGAAGAAGTTTATTTTGCGCGTCGCGCACTGCGTGGA
N004859     (173) CTCAGCTTTACCTTGGTGAGATTGGTTATTCACCACTGTTAACGGCCGAAGAAGAAGTTTATTTTGCGCGTCGCGCACTGCGTGGA
N004859A    (173) CTCAGCTTTACCTTGGTGAGATTGGTTATTCACCACTGTTAACGGCCGAAGAAGAAGTTTATTTTGCGCGTCGCGCACTGCGTGGA
N004859B    (173) CTCAGCTTTACCTTGGTGAGATTGGTTATTCACCACTGTTAACGGCCGAAGAAGAAGTTTATTTTGCGCGTCGCGCACTGCGTGGA
N004859C    (173) CTCAGCTTTACCTTGGTGAGATTGGTTATTCACCACTGTTAACGGCCGAAGAAGAAGTTTATTTTGCGCGTCGCGCACTGCGTGGA
```

Section 4

```
          (259) 259        270       280       290       300       310       320       330         344
EDL933    (259) GATGTCGCCTCTCGCCGCCGGATGATCGAGAGTAACTTGCGTCTGGTGGTAAAAATTGCCCGCCGTTATGGCAATCGTGGTCTGGC
EDL933A   (259) GATGTCGCCTCTCGCCGCCGGATGATCGAGAGTAACTTGCGTCTGGTGGTAAAAATTGCCCGCCGTTATGGCAATCGTGGTCTGGC
EDL933B   (242) GATGTCGCCTCTCGCCGCCGGATGATCGAGAGTAA---------------------------------------------------
EDL933C   (258) GATGTCGCCTCTCGCCGCCGGATGA-------------------------------------------------------------
CL106     (259) GATGTCGCCTCTCGCCGCCGGATGATCGAGAGTAACTTGCGTCTGGTGGTAAAAATTGCCCGCCGTTATGGCAATCGTGGTCTGGC
CL106A    (214) --------------------------------------------------------------------------------------
CL106B    (259) GATGTCGCCTCTCGCCGCCGGATGATCGAGAGTAACTTGCGTCTGGTGGTAAAAATTGCCCGCCGTTATGGCAATCGTGGTCTGGC
CL106C     (91) --------------------------------------------------------------------------------------
EC3377    (259) GATGTCGCCTCTCGCCGCCGGATGATCGAGAGTAACTTGCGTCTGGTGGTAAAAATTGCCCGCCGTTATGGCAATCGTGGTCTGGC
EC3377A   (259) GATGTCGCCTCTCGCCGCCGGATGATCGAGAGTAACTTGCGTCTGGTGGTAAAAATTGCCCGCCGTTATGGCAATCGTGGTCTGGC
EC3377B   (214) --------------------------------------------------------------------------------------
EC3377C   (187) --------------------------------------------------------------------------------------
EC2044    (259) GATGTCGCCTCTCGCCGCCGGATGATCGAGAGTAACTTGCGTCTGGTGGTAAAAATTGCCCGCCGTTATGGCAATCGTGGTCTGGC
EC2044A    (91) --------------------------------------------------------------------------------------
EC2044B   (259) GATGTCGCCTCTCGCCGCCGGATGATCGAGAGTAACTTGCGTCTGGTGGTAAAAATTGCCCGCCGTTATGGCAATCGTGGTCTGGC
EC2044C   (259) GATGTCGCCTCTCGCCGCCGGATGATCGAGAGTAACTTGCGTCTGGTGGTAAAAATTGCCCGCCGTTATGGCAATCGTGGTCTGGC
EC6484    (259) GATGTCGCCTCTCGCCGCCGGATGATCGAGAGTAACTTGCGTCTGGTGGTAAAAATTGCCCGCCGTTATGGCAATCGTGGTCTGGC
EC6484A   (259) GATGTCGCCTCTCGCCGCCGGATGATCGAGAGTAACTTGCGTCTGGTGGTAAAAATTGCCCGCCGTTATGGCAATCGTGGTCTGGC
EC6484B   (259) GATGTCGCCTCTCGCCGCCGGATGATCGAGAGTAACTTGCGTCTGGTGGTAAAAATTGCCCGCCGTTATGGCAATCGTGGTCTGGC
EC6484C   (259) GATGTCGCCTCTCGCCGCCGGATGATCGAGAGTAACTTGCGTCTGGTGGTAAAAATTGCCCGCCGTTATGGCAATCGTGGTCTGGC
N004067   (259) GATGTCGCCTCTCGCCGCCGGATGATCGAGAGTAACTTGCGTCTGGTGGTAAAAATTGCCCGCCGTTATGGCAATCGTGGTCTGGC
N004067A  (259) GATGTCGCCTCTCGCCGCCGGATGATCGAGAGTAACTTGCGTCTGGTGGTAAAAATTGCCCGCCGTTATGGCAATCGTGGTCTGGC
N004067B  (259) GATGTCGCCTCTCGCCGCCGGATGATCGAGAGTAACTTGCGTCTGGTGGTAAAAATTGCCCGCCGTTATGGCAATCGTGGTCTGGC
N004067C  (259) GATGTCGCCTCTCGCCGCCGGATGATCGAGAGTAACTTGCGTCTGGTGGTAAAAATTGCCCGCCGTTATGGCAATCGTGGTCTGGC
N004859   (259) GATGTCGCCTCTCGCCGCCGGATGATCGAGAGTAACTTGCGTCTGGTGGTAAAAATTGCCCGCCGTTATGGCAATCGTGGTCTGGC
N004859A  (259) GATGTCGCCTCTCGCCGCCGGATGATCGAGAGTAACTTGCGTCTGGTGGTAAAAATTGCCCGCCGTTATGGCAATCGTGGTCTGGC
N004859B  (259) GATGTCGCCTCTCGCCGCCGGATGATCGAGAGTAACTTGCGTCTGGTGGTAAAAATTGCCCGCCGTTATGGCAATCGTGGTCTGGC
N004859C  (259) GATGTCGCCTCTCGCCGCCGGATGATCGAGAGTAACTTGCGTCTGGTGGTAAAAATTGCCCGCCGTTATGGCAATCGTGGTCTGGC
```

Section 5

```
                (345) 345  350       360       370       380       390       400       410       420       430
EDL933          (345) GTTGCTGGACC-------------TTATCGAAGAGGGCAACCTGGGGCTGATCCG-TGC----------GGTAGAGAAGTTTGACC
EDL933A         (345) GTTGCTGGACC-------------TTATCGAAGAGGGCAACCTGGGGCTGATCCG-TGCTTTGGTAGAGGGTAGAGAAGTTTGA--
EDL933B         (277) --------------------------------------------------------------------------------------
EDL933C         (283) --------------------------------------------------------------------------------------
CL106           (345) GTTGCTGGACC-------------TTATCGAAGAGGGCAACCTGGGGCTGATCCG-CGC----------GGTAGAGAAGTTTGACC
CL106A          (214) --------------------------------------------------------------------------------------
CL106B          (345) GTTGCTGGACC-------------TTATCGAAGAGGGCAACCTGGGG--GATCCG-CGC----------GGTAGAGAAGTTTGACC
CL106C           (91) --------------------------------------------------------------------------------------
EC3377          (345) GTTGCTGGACC-------------TTATCGAAGAGGGCAACCTGGGGCTGATCCG-CGC----------GGTAGAGAAGTTTGACC
EC3377A         (345) GTTGCTGGACCGCGTTGCTGGACCTTATCGAAGAGGGCAACCTGGGGCTGATCCG-CGC----------GGTAGAGAAGTTTGA--
EC3377B         (214) --------------------------------------------------------------------------------------
EC3377C         (187) --------------------------------------------------------------------------------------
EC2044          (345) GTTGCTGGACC-------------TTATCGAAGAGGGCAACCTGGGGCTGATCCG-CGC----------GGTAGAGAAGTTTGACC
EC2044A          (91) --------------------------------------------------------------------------------------
EC2044B         (345) GTTGCTGGACC-------------TTATCGAAGAGGGCAACCTGGGGCTGATCCG-CGC----------GGTAGAGAAGTTTGACC
EC2044C         (345) GTTGCTGGACC-------------TTATCGAAGAGGGCAACCTGGGGCTGATCCG-CGC----------GGTAGAGAAGTTTGACC
EC6484          (345) GTTGCTGGACC-------------TTATCGAAGAGGGCAACCTGGGGCTGATCCG-CGC----------GGTAGAGAAGTTTGACC
EC6484A         (345) GTTGCTGGACC-------------TTATCGAAGAGGGCAACCTGGGGCTGATCCG-CGC----------GGTAGAGAAGTTTGACC
EC6484B         (345) GTTGCTGGACC-------------TTATCGAAGAGGGCAACCTGGGGCTGATCCG-CGC----------GGTAGAGAAGTTTGACC
EC6484C         (345) GTTGCTGGACC-------------TTATCGAAGAGGGCAACCTGGGGCTGATCCGGCGC----------GGTAGAGAAGTTTGA--
N004067         (345) GTTGCTGGACC-------------TTATCGAAGAGGGCAACCTGGGGCTGATCCG-CGC----------GGTAGAGAAGTTTGACC
N004067A        (345) GTTGCTGGACC-------------TTATCGAAGAGGGCAACCTGGGGCTGATCCG-CGC----------GGTAGAGAAGTTTGACC
N004067B        (345) GTTGCTGGACC-------------TTATCGAAGAGGGCAACCTGGGGCTGATCCG-CGC----------GGTAGAGAAGTTTGACC
N004067C        (345) GTTGCTGGACC-------------TTATCGAAGAGGGCAACCTGGGGCTGATCCG-CGC----------GGTAGAGAAGTTTGACC
N004859         (345) GTTGCTGGACC-------------TTATCGAAGAGGGCAACCTGGGGCTGATCCG-CGC----------GGTAGAGAAGTTTGACC
N004859A        (345) GTTGCTGGACC-------------TTATCGAAGAGGGCAACCTGGGGCTGATCCG-CGC----------GGTAGAGAAGTTTGACC
N004859B        (345) GTTGCTGGACC-------------TTATCGAAGAGGGCAACCTGGGGCTGATCCG-CGC----------GGTAGAGAAGTTTGACC
N004859C        (345) GTTGCTGGACC-------------TTATCGAAGAGGGCAACCTGGGGCTGATCCG-CGC----------GGTAGAGAAGTTTGACC
```

Section 6

```
          (431) 431      440       450       460       470       480       490       500            516
   EDL933 (407) CGGAACGTGGTTTCCGCTTCTCAACATACGCAACCTGGTGGATTCGCCAGACGATTGAACGGGCGATTATGAACCAAACCCGTACT
  EDL933A (415) --------------------------------------------------------------------------------------
  EDL933B (277) --------------------------------------------------------------------------------------
  EDL933C (283) --------------------------------------------------------------------------------------
    CL106 (407) CGGAACGTGGTTTCCGCTTCTCAACATACGCAACCTGGTGGATTCGCCAGACGATTGAACGGGCGATTATGAACCAAACCCGTACT
   CL106A (214) --------------------------------------------------------------------------------------
   CL106B (405) CGGAACGTGGTTTCCGCTTCTCAACATACGCAACCTGGTGGATTCGCCAGACGATTGA----------------------------
   CL106C  (91) --------------------------------------------------------------------------------------
   EC3377 (407) CGGAACGTGGTTTCCGCTTCTCAACATACGCAACCTGGTGGATTCGCCAGACGATTGAACGGGCGATTATGAACCAAACCCGTACT
  EC3377A (418) --------------------------------------------------------------------------------------
  EC3377B (214) --------------------------------------------------------------------------------------
  EC3377C (187) --------------------------------------------------------------------------------------
   EC2044 (407) CGGAACGTGGTTTCCGCTTCTCAACATACGCAACCTGGTGGATTCGCCAGACGATTGAACGGGCGATTATGAACCAAACCCGTACT
  EC2044A  (91) --------------------------------------------------------------------------------------
  EC2044B (407) CGGAACGTGGTTTCCGCTTCTCAACATACGCAACCTGGTGGATTCGCCAGACGATTGAACGGGCGATTATGAACCAAACCCGTACT
  EC2044C (407) CGGAACGTGGTTTCCGCTTCTCAACATACGCAACCTGGTGGATTCGCCAGACGATTGAACGGGCGATTATGAACCAAACCCGTACT
   EC6484 (407) CGGAACGTGGTTTCCGCTTCTCAACATACGCAACCTGGTGGATTCGCCAGACGATTGAACGGGCGATTATGAACCAAACCCGTACT
  EC6484A (407) CGGAACGTGGTTTCCGCTTCTCAACATACGCAACCTGGTGGATTCGCCAGACGATTGAACGGGCGATTATGAACCAAACCCGTACT
  EC6484B (407) CGGAACGTGGTTTCCGCTTCTCAACATACGCAACCTGGTGGATTCGCCAGACGATTGAACGGGCGATTATGAACCAAACCCGTACT
  EC6484C (406) --------------------------------------------------------------------------------------
  N004067 (407) CGGAACGTGGTTTCCGCTTCTCAACATACGCAACCTGGTGGATTCGCCAGACGATTGAACGGGCGATTATGAACCAAACCCGTACT
 N004067A (407) CGGAACGTGGTTTCCGCTTCTCAACATACGCAACCTGGTGGATTCGCCAGACGATTGAACGGGCGATTATGAACCAAACCCGTACT
 N004067B (407) CGGAACGTGGTTTCCGCTTCTCAACATACGCAACCTGGTGGATTCGCCAGACGATTGAACGGGCGATTATGAACCAAACCCGTACT
 N004067C (407) CGGAACGTGGTTTCCGCTTCTCAACATACGCAACCTGGTGGATTCGCCAGACGATTGAACGGGCGATTATGAACCAAACC-GTACT
  N004859 (407) CGGAACGTGGTTTCCGCTTCTCAACATACGCAACCTGGTGGATTCGCCAGACGATTGAACGGGCGATTATGAACCAAACCCGTACT
 N004859A (407) CGGAACGTGGTTTCCGCTTCTCAACATACGCAACCTGGTGGATTCGCCAGACGATTGAACGGGCGATTATGAACCAAACCCGTACT
 N004859B (407) CGGAACGTGGTTTCCGCTTCTCAACATACGCAACCTGGTGGATTCGCCAGACGATTGAACGGGCGATTATGAACCAAACCCGTACT
 N004859C (407) CGGAACGTGGTTTCCGCTTCTCAACATACGCAACCTGGTGGATTCGCCAGACGATTGAACGGGCG-TTATGA--------------
```

```
           (517) 517          530       540       550       560       570       580       590        602
EDL933     (493) ATTCGTTTGCCGATTCACATCGTAAAGGAGCTGAAC-GTTTACCTGCGAACAGCACGTGAGTTGTCCCATAAGCTGGACCATGAAC
EDL933A    (415) --------------------------------------------------------------------------------------
EDL933B    (277) --------------------------------------------------------------------------------------
EDL933C    (283) --------------------------------------------------------------------------------------
CL106      (493) ATTCGTTTGCCGATTCACATCGTAAAGGAGCTGAAC-GTTTACCTGCGAACCGCACGTGAGTTGTCCCATAAGCTGGACCATGAAC
CL106A     (214) --------------------------------------------------------------------------------------
CL106B     (463) --------------------------------------------------------------------------------------
CL106C      (91) --------------------------------------------------------------------------------------
EC3377     (493) ATTCGTTTGCCGATTCACATCGTAAAGGAGCTGAAC-GTTTACCTGCGAACCGCACGTGAGTTGTCCCATAAGCTGGACCATGAAC
EC3377A    (418) --------------------------------------------------------------------------------------
EC3377B    (214) --------------------------------------------------------------------------------------
EC3377C    (187) --------------------------------------------------------------------------------------
EC2044     (493) ATTCGTTTGCCGATTCACATCGTAAAGGAGCTGAAC-GTTTACCTGCGAACCGCACGTGAGTTGTCCCATAAGCTGGACCATGAAC
EC2044A     (91) --------------------------------------------------------------------------------------
EC2044B    (493) ATTCGTTTGCCGATTCACATCGTAAAGGAGCTGAAC-GTTTACCTGCGAACCGCACGTGAGTTGTCCCATAAGCTGGACCATGAAC
EC2044C    (493) ATTCGTTTGCCGATTCACATCGTAAAGGAGCTGAAC-GTTTACCTGCGAACCGCACGTGAGTTGTCCCATAAGCTGGACCATGAAC
EC6484     (493) ATTCGTTTGCCGATTCACATCGTAAAGGAGCTGAAC-GTTTACCTGCGAACCGCACGTGAGTTGTCCCATAAGCTGGACCATGAAC
EC6484A    (493) ATTCGTTTGCCGATTCACATCGTAAAGGAGCTGAAC-GTTTACCTGCGAACCGCACGTGAGTTGTCCCATAAGCTGGACCATGAAC
EC6484B    (493) ATTCGTTTGCCGATTCACATCGTAAAGGAGCTGAAC-GTTTACCTGCGAACCGCACGTGAGTTGTCCCATAAGCTGGACCATGAAC
EC6484C    (406) --------------------------------------------------------------------------------------
N004067    (493) ATTCGTTTGCCGATTCACATCGTAAAGGAGCTGAAC-GTTTACCTGCGAACCGCACGTGAGTTGTCCCATAAGCTGGACCATGAAC
N004067A   (493) ATTCGTTTGCCGATTCACATCGTAAAGGAGCTGAAC-GTTTACCTGCGAACCGCACGTGAGTTGTCCCATAAGCTGGACCATGAAC
N004067B   (493) ATTCGTTTGCCGATTCACATCGTAAAGGAGCTGAAC-GTTTACCTGCGAACCGCACGTGAGTTGTCCCATAAGCTGGACCATGAAC
N004067C   (492) ATTCGTTTGCCGATTCACATCGTAA-------------------------------------------------------------
N004859    (493) ATTCGTTTGCCGATTCACATCGTAAAGGAGCTGAAC-GTTTACCTGCGAACCGCACGTGAGTTGTCCCATAAGCTGGACCATGAAC
N004859A   (493) ATTCGTTTGCCGATTCACATCGTAAAGGAGCTGAAC-GTTTACCTGCGAACCGCACGTGAGTTGTCCCATAAGCTGGACCATGAAC
N004859B   (493) ATTCGTTTGCCGATTCACATCGTAAAGGAGCTGAACCGTTTACCTGCGAACCGCACGTGA--------------------------
N004859C   (478) --------------------------------------------------------------------------------------
```

Section 8

	(603)	603 610 620 630 640 650 660 670 688
EDL933	(578)	CAAGTGCGGAAGAGATCGCAGAGCAACTGGATAAGCCAGTTGATGACGTCAGCCGTATGCTTCGTCTTAACGAGCGCATTACCTCG
EDL933A	(415)	--
EDL933B	(277)	--
EDL933C	(283)	--
CL106	(578)	CAAGTGCGGAAGAGATCGCAGAGCAACTGGATAAGCCAGTTGATGACGTCAGCCGTATGCTTCGTCTTAACGAGCGCATTACCTCG
CL106A	(214)	--
CL106B	(463)	--
CL106C	(91)	--
EC3377	(578)	CAAGTGCGGAAGAGATCGCAGAGCAACTGGATAAGCCAGTTGATGACGTCAGCCGTATGCTTCGTCTTAACGAGCGCATTACCTCG
EC3377A	(418)	--
EC3377B	(214)	--
EC3377C	(187)	--
EC2044	(578)	CAAGTGCGGAAGAGATCGCAGAGCAACTGGATAAGCCAGTTGATGACGTCAGCCGTATGCTTCGTCTTAACGAGCGCATTACCTCG
EC2044A	(91)	--
EC2044B	(578)	CAAGTGCGGAAGAGATCGCAGAGCAACTGGATAAGCCAGTTGATGACGTCAGCCGTATGCTTCGTCTTAACGAGCGCATTACCTCG
EC2044C	(578)	CAAGTGCGGAAGAGATCGCAGAGCAACTGGATAAGCCAGTTGATGACGTCAGCCGTATGCTTCGTCTTAACGAGCGCATTACCTCG
EC6484	(578)	CAAGTGCGGAAGAGATCGCAGAGCAACTGGATAAGCCAGTTGATGACGTCAGCCGTATGCTTCGTCTTAACGAGCGCATTACCTCG
EC6484A	(578)	CAAGTGCGGAAGAGATCGCAGAGCAACTGGATAAGCCAGTTGATGACGTCAGCCGTATGCTTCGTCTTAACGAGCGCATTACCTCG
EC6484B	(578)	CAAGTGCGGAAGAGATCGCAGAGCAACTGGATAAGCCAGTTGATGACGTCAGCCGTATGCTTCGTCTTAACGAGCGCATTACCTCG
EC6484C	(406)	--
N004067	(578)	CAAGTGCGGAAGAGATCGCAGAGCAACTGGATAAGCCAGTTGATGACGTCAGCCGTATGCTTCGTCTTAACGAGCGCATTACCTCG
N004067A	(578)	CAAGTGCGGAAGAGATCGCAGAGCAACTGGATAAGCCAGTTGATGACGTCAGCCGTATGCTTCGTCTTAACGAGCGCATTACCTCG
N004067B	(578)	CAAGTGCGGAAGAGATCGCAGAGCAACTGGATAAGCCAGTTGATGACGTCAGCCGTATGCTTCGTCTTAACGAGCGCATTACCTCG
N004067C	(517)	--
N004859	(578)	CAAGTGCGGAAGAGATCGCAGAGCAACTGGATAAGCCAGTTGATGACGTCAGCCGTATGCTTCGTCTTAACGAGCGCATTACCTCG
N004859A	(578)	CAAGTGCGGAAGAGATCGCAGAGCAACTGGATAAGCCAGTTGATGACGTCAGCCGTATGCTTCGTCTTAACGAGCGCATTACCTCG
N004859B	(553)	--
N004859C	(478)	--

Section 9

```
           (689) 689        700       710       720       730       740       750       760          774
EDL933     (664) GTAGACACCCCGCTGGGTGGTGATTCCGAAAAAGCGTTGCTGGACATCCTGGCCGATGAAAAAGAGAACGGTCCGGAAGATA--CC
EDL933A    (415) --------------------------------------------------------------------------------------
EDL933B    (277) --------------------------------------------------------------------------------------
EDL933C    (283) --------------------------------------------------------------------------------------
CL106      (664) GTAGACACCCCGCTGGGTGGTGATTCCGAAAAAGCGTTGCTGGACATCCTGGCCGATGAAAAAGAGAACGGTCCGGAAGATA--CC
CL106A     (214) --------------------------------------------------------------------------------------
CL106B     (463) --------------------------------------------------------------------------------------
CL106C      (91) --------------------------------------------------------------------------------------
EC3377     (664) GTAGACACCCCGCTGGGTGGTGATTCCGAAAAAGCGTTGCTGGACATCCTGGCCGATGAAAAAGAGAACGGTCCGGAAGATA--CC
EC3377A    (418) --------------------------------------------------------------------------------------
EC3377B    (214) --------------------------------------------------------------------------------------
EC3377C    (187) --------------------------------------------------------------------------------------
EC2044     (664) GTAGACACCCCGCTGGGTGGTGATTCCGAAAAAGCGTTGCTGGACATCCTGGCCGATGAAAAAGAGAACGGTCCGGAAGATA--CC
EC2044A     (91) --------------------------------------------------------------------------------------
EC2044B    (664) GTAGACACCCCGCTGGGTGGTGATTCCGAAAAAGCGTTGCTGGACATCCTGGCCGATGAAAAATAG--------------------
EC2044C    (664) GTAGACACCCCGCTGGGTGGTGATTCCGAAAAAGCGTTGCTGGACATCCTGGCCGATGAAAAAGAGAACGGTCCGGAAGATA--CC
EC6484     (664) GTAGACACCCCGCTGGGTGGTGATTCCGAAAAAGCGTTGCTGGACATCCTGGCCGATGAAAAAGAGAACGGTCCGGAAGATA--CC
EC6484A    (664) GTAGACACCCCGCTGGGTGGTGATTCCGAAAAAGCGTTGCTGGACATCCTGGCCGATGAAAAAGAGAACGGTCCGGAAGATATACC
EC6484B    (664) GTAGACACCCCGCTGGGTGGTGATTCCGAAAAAGCGTTGCTGGACATCCTGGCCGATGAAAAAGAGAACGGTCCGGAAGATA--CC
EC6484C    (406) --------------------------------------------------------------------------------------
N004067    (664) GTAGACACCCCGCTGGGTGGTGATTCCGAAAAAGCGTTGCTGGACATCCTGGCCGATGAAAAAGAGAACGGTCCGGAAGATA--CC
N004067A   (664) GTAGACACCCCGCTGGGTGGTGATTCCGAAAAAGCGTTGCTGGACATCCTGGCCGATGAAAAAGAGAACGGTCCGGAA---ATACC
N004067B   (664) GTAGACACCCCGCTGGGTGGTGATTCCGAAAAAGCGTTGCTGGACATCCTGGCCGATGAAAAAGAGAACGGTCCGGAAGATA--CC
N004067C   (517) --------------------------------------------------------------------------------------
N004859    (664) GTAGACACCCCGCTGGGTGGTGATTCCGAAAAAGCGTTGCTGGACATCCTGGCCGATGAAAAAGAGAACGGTCCGGAAGATA--CC
N004859A   (664) GTAGACACCCCGCTGGGTGGTGATTCCGAAAAAGCGTTGCTGGACATCCTGGCCGATGAAAAAGAGAACGGTCCGGAAGATA--CC
N004859B   (553) --------------------------------------------------------------------------------------
N004859C   (478) --------------------------------------------------------------------------------------
```

Section 10

```
              (775) 775  780       790       800       810       820       830       840       850       860
EDL933        (748) ACGCAAGATGACGATATGAAGCAGAGCATCGTCAAATGGCTGTTCGAGCTGAACGCCAAACAGCGTGAAGTACTGGCACGTCGATT
EDL933A       (415) --------------------------------------------------------------------------------------
EDL933B       (277) --------------------------------------------------------------------------------------
EDL933C       (283) --------------------------------------------------------------------------------------
CL106         (748) ACGCAAGATGACGATATGAAGCAGAGCATCGTCAAATGGCTGTTCGAGCTGAACGCCAAACAGCGTGAAGTACTGGCACGTCGATT
CL106A        (214) --------------------------------------------------------------------------------------
CL106B        (463) --------------------------------------------------------------------------------------
CL106C         (91) --------------------------------------------------------------------------------------
EC3377        (748) ACGCAAGATGACGATATGAAGCAGAGCATCGTCAAATGGCTGTTCGAGCTGAACGCCAAACAGCGTGAAGTGCTGGCACGTCGATT
EC3377A       (418) --------------------------------------------------------------------------------------
EC3377B       (214) --------------------------------------------------------------------------------------
EC3377C       (187) --------------------------------------------------------------------------------------
EC2044        (748) ACGCAAGATGACGATATGAAGCAGAGCATCGTCAAATGGCTGTTCGAGCTGAACGCCAAACAGCGTGAAGTGCTGGCACGTCGATT
EC2044A        (91) --------------------------------------------------------------------------------------
EC2044B       (730) --------------------------------------------------------------------------------------
EC2044C       (748) ACGCAAGATGACGATATGAAGCAGAGCATCGTCAAATGGCTGTTCGAGCTGAACGCCAAACAGCGTGAAGTGCTGGCACGTCGATT
EC6484        (748) ACGCAAGATGACGATATGAAGCAGAGCATCGTCAAATGGCTGTTCGAGCTGAACGCCAAACAGCGTGAAGTGCTGGCACGTCGATT
EC6484A       (750) ACGCAAGATGACGATATGA-------------------------------------------------------------------
EC6484B       (748) ACGCAAGATGACGATATGAAGCAGAGCATCGTCAAATGGCTGTTCGAGCTGAACGCCAAACAGCGTGAAGTGCTGGCACGTTGA--
EC6484C       (406) --------------------------------------------------------------------------------------
N004067       (748) ACGCAAGATGACGATATGAAGCAGAGCATCGTCAAATGGCTGTTCGAGCTGAACGCCAAACAGCGTGAAGTGCTGGCACGTCGATT
N004067A      (747) ACGCAAGATGACGATATGA-------------------------------------------------------------------
N004067B      (748) ACGCAAGATGACGATATGAAGCAGAGCATCGTCAAATGGCTGTTCGAGCTGAACGCCAAACAGCGTGAAGTGCTGGCACGTCGATT
N004067C      (517) --------------------------------------------------------------------------------------
N004859       (748) ACGCAAGATGACGATATGAAGCAGAGCATCGTCAAATGGCTGTTCGAGCTGAACGCCAAACAGCGTGAAGTGCTGGCACGTCGATT
N004859A      (748) ACGCAAGATGACGATATGAAGCAGAGCATCGTCAAATGGCTGTTCGAGCTGAACGCCAAACAGCGTGAAGTGCTGGCACGTCGATT
N004859B      (553) --------------------------------------------------------------------------------------
N004859C      (478) --------------------------------------------------------------------------------------
```

```
          (861) 861      870       880       890       900       910       920       930            946
EDL933    (834) CGGTTTGCTGGGGTACGAAGCGGCAACAC-TGGAAGATGTAGGTCGTGAAATTGGCCTCACCCGTGAACGTGTTCGCCAGATTCAG
EDL933A   (415) --------------------------------------------------------------------------------------
EDL933B   (277) --------------------------------------------------------------------------------------
EDL933C   (283) --------------------------------------------------------------------------------------
CL106     (834) CGGTTTGCTGGGGTACGAAGCGGCAACAC-TGGAAGATGTAGGTCGTGAAATTGGCCTCACCCGTGAACGTGTTCGCCAGATTCAG
CL106A    (214) --------------------------------------------------------------------------------------
CL106B    (463) --------------------------------------------------------------------------------------
CL106C     (91) --------------------------------------------------------------------------------------
EC3377    (834) CGGTTTGCTGGGGTACGAAGCGGCAACAC-TGGAAGATGTAGGTCGTGAAATTGGCCTCACCCGTGAACGTGTTCGCCAGATTCAG
EC3377A   (418) --------------------------------------------------------------------------------------
EC3377B   (214) --------------------------------------------------------------------------------------
EC3377C   (187) --------------------------------------------------------------------------------------
EC2044    (834) CGGTTTGCTGGGGTACGAAGCGGCAACAC-TGGAAGATGTAGGTCGTGAAATTGGCCTCACCCGTGAACGTGTTCGCCAGATTCAG
EC2044A    (91) --------------------------------------------------------------------------------------
EC2044B   (730) --------------------------------------------------------------------------------------
EC2044C   (834) CGGTTTGCTGGGGTAG----------------------------------------------------------------------
EC6484    (834) CGGTTTGCTGGGGTACGAAGCGGCAACAC-TGGAAGATGTAGGTCGTGAAATTGGCCTCACCCGTGAACGTGTTCGCCAGATTCAG
EC6484A   (769) --------------------------------------------------------------------------------------
EC6484B   (832) --------------------------------------------------------------------------------------
EC6484C   (406) --------------------------------------------------------------------------------------
N004067   (834) CGGTTTGCTGGGGTACGAAGCGGCAACAC-TGGAAGATGTAGGTCGTGAAATTGGCCTCACCCGTGAACGTGTTCGCCAGATTCAG
N004067A  (766) --------------------------------------------------------------------------------------
N004067B  (834) CGGTTTGCTGGGGTACGAAGCGGCAACAC-TGGAAGATGTAGGTCGTGAAATTGGCCTCACCCGTGAACGTGTTCGCCAGATTCAG
N004067C  (517) --------------------------------------------------------------------------------------
N004859   (834) CGGTTTGCTGGGGTACGAAGCGGCAACAC-TGGAAGATGTAGGTCGTGAAATTGGCCTCACCCGTGAACGTGTTCGCCAGATTCAG
N004859A  (834) CGGTTTGCTGGGGTACGAAGCGGCAACACGTGGAAGATGTAGGTCGTGA-------------------------------------
N004859B  (553) --------------------------------------------------------------------------------------
N004859C  (478) --------------------------------------------------------------------------------------
```

Section 12

```
                  (947) 947          960       970       980       990       1000      1010      1020        1032
EDL933            (919) GTTGAAGGCCTGCGCCGTTTGCGCGAAATCCTGCAAACGCAGGGGCTGAATATCGAAGCGCTGTTCCGCGAGTAA-----------
EDL933A           (415) --------------------------------------------------------------------------------------
EDL933B           (277) --------------------------------------------------------------------------------------
EDL933C           (283) --------------------------------------------------------------------------------------
CL106             (919) GTTGAAGGCCTGCGCCGTTTGCGCGAAATCCTGCAAACGCAGGGGCTGAATATCGAAGCGCTGTTCCGCGAGTAA-----------
CL106A            (214) --------------------------------------------------------------------------------------
CL106B            (463) --------------------------------------------------------------------------------------
CL106C             (91) --------------------------------------------------------------------------------------
EC3377            (919) GTTGAAGGCCTGCGCCGTTTGCGCGAAATCCTGCAAACGCAGGGGCTGAATATCGAAGCGCTGTTCCGCGAGTAA-----------
EC3377A           (418) --------------------------------------------------------------------------------------
EC3377B           (214) --------------------------------------------------------------------------------------
EC3377C           (187) --------------------------------------------------------------------------------------
EC2044            (919) GTTGAAGGCCTGCGCCGTTTGCGCGAAATCCTGCAAACGCAGGGGCTGAATATCGAAGCGCTGTTCCGCGAGTAA-----------
EC2044A            (91) --------------------------------------------------------------------------------------
EC2044B           (730) --------------------------------------------------------------------------------------
EC2044C           (850) --------------------------------------------------------------------------------------
EC6484            (919) GTTGAAGGCCTGCGCCGTTTGCGCGAAATCCTACAAACGCAGGGGCTGAATATCGAAGCGCTGTTCCGCGAGTAA-----------
EC6484A           (769) --------------------------------------------------------------------------------------
EC6484B           (832) --------------------------------------------------------------------------------------
EC6484C           (406) --------------------------------------------------------------------------------------
N004067           (919) GTTGAAGGCCTGCGCCGTTTGCGCGAAATCCTACAAACGCAGGGGCTGAATATCGAAGCGCTGTTCCGCGAGTAA-----------
N004067A          (766) --------------------------------------------------------------------------------------
N004067B          (919) GTTTAA--------------------------------------------------------------------------------
N004067C          (517) --------------------------------------------------------------------------------------
N004859           (919) GTTGAAGGCCTGCGCCGTTTGCGCGAAATCCTGCAAACGCAGGGGCTGAATATCGAAGCGCTGTTCCGCGAGTAA-----------
N004859A          (883) --------------------------------------------------------------------------------------
N004859B          (553) --------------------------------------------------------------------------------------
N004859C          (478) --------------------------------------------------------------------------------------
```

	(1033)	1033 1045
EDL933	(994)	-------------
EDL933A	(415)	-------------
EDL933B	(277)	-------------
EDL933C	(283)	-------------
CL106	(994)	-------------
CL106A	(214)	-------------
CL106B	(463)	-------------
CL106C	(91)	-------------
EC3377	(994)	-------------
EC3377A	(418)	-------------
EC3377B	(214)	-------------
EC3377C	(187)	-------------
EC2044	(994)	-------------
EC2044A	(91)	-------------
EC2044B	(730)	-------------
EC2044C	(850)	-------------
EC6484	(994)	-------------
EC6484A	(769)	-------------
EC6484B	(832)	-------------
EC6484C	(406)	-------------
N004067	(994)	-------------
N004067A	(766)	-------------
N004067B	(925)	-------------
N004067C	(517)	-------------
N004859	(994)	-------------
N004859A	(883)	-------------
N004859B	(553)	-------------
N004859C	(478)	-------------
