# Supplementary material for: Polymorphism and selection of rpoS in pathogenic Escherichia coli
Source: BMC Microbiol. 2009 Jun 3;9:118. doi: 10.1186/1471-2180-9-118 (PMC2700278; doi:10.1186/1471-2180-9-118)
Supplement: Additional file 2 — Alignment of predicted RpoS protein sequences of Suc++ mutants with parental strains. The protein alignment data show the predicted mutant forms of RpoS resulting from the identified mutations in the rpoS gene of Suc++ mutants. [file 1471-2180-9-118-S2.pdf]

Section 1

```
                1        10        20        30        40        50        60        70              87
            (1) 
EDL933      (1) MSQNTLKVHDLNEDAEFDENGVEVFDEKALVEEEPSDNDLAEEELLSQGATQRVLDATQLYLGEIGYSPLLTAEEEVYFARRALRGD
EDL933A     (1) MSQNTLKVHDLNEDAEFDENGVEVFDEKALVEEEPSDNDLAEEELLSQGATQRVLDATQLYLGEIGYSPLLTAEEEVYFARRALRGD
EDL933B     (1) MSQNTLKVHDLNEDAEFDENGVEVFDEKALVEEEPSDNDLAEEELLSQGATQRVLDATQLYLTTVNGRRRSLFCASRTAWRCRLSPP
EDL933C     (1) MSQNTLKVHDLNEDAEFDENGVEVFDEKALVEEEPSDNDLAEEELLSQGATQRVLDATQLYLGEIGYSPLLTAEKKFILRVAHCVEM
CL106       (1) MSQNTLKVHDLNEDAEFDENGVEVFDEKALVEEEPSDNDLAEEELLSQGATQRVLDATQLYLGEIGYSPLLTAEEEVYFARRALRGD
CL106A      (1) MSQNTLKVHDLNEDAEFDENGVEVFDEKALVEEEPSDNDLAEEELLSQGATQRVLDATQLYLVRLVIHHC-----------------
CL106B      (1) MSQNTLKVHDLNEDAEFDENGVEVFDEKALVEEEPSDNDLAEEELLSQGATQRVLDATQLYLGEIGYSPLLTAEEEVYFARRALRGD
CL106C      (1) MSQNTLKVHDLNEDAEFDENGVEVFDEKA----------------------------------------------------------
EC3377      (1) MSQNTLKVHDLNEDAEFDENGVEVFDEKALVEEEPSDNDLAEEELLSQGATQRVLDATQLYLGEIGYSPLLTAEEEVYFARRALRGD
EC3377A     (1) MSQNTLKVHDLNEDAEFDENGVEVFDEKALVEEEPSDNDLAEEELLSQGATQRVLDATQLYLGEIGYSPLLTAEEEVYFARRALRGD
EC3377B     (1) MSQNTLKVHDLNEDAEFDENGVEVFDEKALVEEEPSDNDLAEEELLSQGAHSVCWTRLSFTLVRLVIHHC-----------------
EC3377C     (1) MSQNTLKVHDLNEDAEFDENGVEVFDEKALVEEEPSDNDLAEEELLSQGAACVGRDSALPW--------------------------
EC2044      (1) MSQNTLKVHDLNEDAEFDENGVEVFDEKALVEEEPSDNDLAEEELLSQGATQRVLDATQLYLGEIGYSPLLTAEEEVYFARRALRGD
EC2044A     (1) MSQNTLKVHDLNEDAEFDENGVEVLTKRP----------------------------------------------------------
EC2044B     (1) MSQNTLKVHDLNEDAEFDENGVEVFDEKALVEEEPSDNDLAEEELLSQGATQRVLDATQLYLGEIGYSPLLTAEEEVYFARRALRGD
EC2044C     (1) MSQNTLKVHDLNEDAEFDENGVEVFDEKALVEEEPSDNDLAEEELLSQGATQRVLDATQLYLGEIGYSPLLTAEEEVYFARRALRGD
EC6484      (1) MSQNTLKVHDLNEDAEFDENGVEVFDEKALVEEEPSDNDLAEEELLSQGATQRVLDATQLYLGEIGYSPLLTAEEEVYFARRALRGD
EC6484A     (1) MSQNTLKVHDLNEDAEFDENGVEVFDEKALVEEEPSDNDLAEEELLSQGATQRVLDATQLYLGEIGYSPLLTAEEEVYFARRALRGD
EC6484B     (1) MSQNTLKVHDLNEDAEFDENGVEVFDEKALVEEEPSDNDLAEEELLSQGATQRVLDATQLYLGEIGYSPLLTAEEEVYFARRALRGD
EC6484C     (1) MSQNTLKVHDLNEDAEFDENGVEVFDEKALVEEEPSDNDLAEEELLSQGATQRVLDATQLYLGEIGYSPLLTAEEEVYFARRALRGD
N004067     (1) MSQNTLKVHDLNEDAEFDENGVEVFDEKALVEEEPSDNDLAEEELLSQGATQRVLDATQLYLGEIGYSPLLTAEEEVYFARRALRGD
N004067A    (1) MSQNTLKVHDLNEDAEFDENGVEVFDEKALVEEEPSDNDLAEEELLSQGATQRVLDATQLYLGEIGYSPLLTAEEEVYFARRALRGD
N004067B    (1) MSQNTLKVHDLNEDAEFDENGVEVFDEKALVEEEPSDNDLAEEELLSQGATQRVLDATQLYLGEIGYSPLLTAEEEVYFARRALRGD
N004067C    (1) MSQNTLKVHDLNEDAEFDENGVEVFDEKALVEEEPSDNDLAEEELLSQGATQRVLDATQLYLGEIGYSPLLTAEEEVYFARRALRGD
N004859     (1) MSQNTLKVHDLNEDAEFDENGVEVFDEKALVEEEPSDNDLAEEELLSQGATQRVLDATQLYLGEIGYSPLLTAEEEVYFARRALRGD
N004859A    (1) MSQNTLKVHDLNEDAEFDENGVEVFDEKALVEEEPSDNDLAEEELLSQGATQRVLDATQLYLGEIGYSPLLTAEEEVYFARRALRGD
N004859B    (1) MSQNTLKVHDLNEDAEFDENGVEVFDEKALVEEEPSDNDLAEEELLSQGATQRVLDATQLYLGEIGYSPLLTAEEEVYFARRALRGD
N004859C    (1) MSQNTLKVHDLNEDAEFDENGVEVFDEKALVEEEPSDNDLAEEELLSQGATQRVLDATQLYLGEIGYSPLLTAEEEVYFARRALRGD
```

Section 2

```
          (88) 88          100       110       120       130       140       150       160           174
EDL933    (88) VASRRRMIESNLRLVVKIARRYGNRGLALLDLIEEGNLGLIRAVEKFDPERGFRFSTYATWWIRQTIERAIMNQTRTIRLPIHIVKE
EDL933A   (88) VASRRRMIESNLRLVVKIARRYGNRGLALLDLIEEGNLGLIRALVEGREV-------------------------------------
EDL933B   (88) DDRE-----------------------------------------------------------------------------------
EDL933C   (88) SPLAAG---------------------------------------------------------------------------------
CL106     (88) VASRRRMIESNLRLVVKIARRYGNRGLALLDLIEEGNLGLIRAVEKFDPERGFRFSTYATWWIRQTIERAIMNQTRTIRLPIHIVKE
CL106A    (71) ---------------------------------------------------------------------------------------
CL106B    (88) VASRRRMIESNLRLVVKIARRYGNRGLALLDLIEEGNLGDPRGRE-----------VPGTWFPLLNIRNLVDSPDD-----------
CL106C    (30) ---------------------------------------------------------------------------------------
EC3377    (88) VASRRRMIESNLRLVVKIARRYGNRGLALLDLIEEGNLGLIRAVEKFDPERGFRFSTYATWWIRQTIERAIMNQTRTIRLPIHIVKE
EC3377A   (88) VASRRRMIESNLRLVVKIARRYGNRGLALLDRVAGPYRRGQPGADPRGREV------------------------------------
EC3377B   (71) ---------------------------------------------------------------------------------------
EC3377C   (62) ---------------------------------------------------------------------------------------
EC2044    (88) VASRRRMIESNLRLVVKIARRYGNRGLALLDLIEEGNLGLIRAVEKFDPERGFRFSTYATWWIRQTIERAIMNQTRTIRLPIHIVKE
EC2044A   (30) ---------------------------------------------------------------------------------------
EC2044B   (88) VASRRRMIESNLRLVVKIARRYGNRGLALLDLIEEGNLGLIRAVEKFDPERGFRFSTYATWWIRQTIERAIMNQTRTIRLPIHIVKE
EC2044C   (88) VASRRRMIESNLRLVVKIARRYGNRGLALLDLIEEGNLGLIRAVEKFDPERGFRFSTYATWWIRQTIERAIMNQTRTIRLPIHIVKE
EC6484    (88) VASRRRMIESNLRLVVKIARRYGNRGLALLDLIEEGNLGLIRAVEKFDPERGFRFSTYATWWIRQTIERAIMNQTRTIRLPIHIVKE
EC6484A   (88) VASRRRMIESNLRLVVKIARRYGNRGLALLDLIEEGNLGLIRAVEKFDPERGFRFSTYATWWIRQTIERAIMNQTRTIRLPIHIVKE
EC6484B   (88) VASRRRMIESNLRLVVKIARRYGNRGLALLDLIEEGNLGLIRAVEKFDPERGFRFSTYATWWIRQTIERAIMNQTRTIRLPIHIVKE
EC6484C   (88) VASRRRMIESNLRLVVKIARRYGNRGLALLDLIEEGNLGLIRRGREV----------------------------------------
N004067   (88) VASRRRMIESNLRLVVKIARRYGNRGLALLDLIEEGNLGLIRAVEKFDPERGFRFSTYATWWIRQTIERAIMNQTRTIRLPIHIVKE
N004067A  (88) VASRRRMIESNLRLVVKIARRYGNRGLALLDLIEEGNLGLIRAVEKFDPERGFRFSTYATWWIRQTIERAIMNQTRTIRLPIHIVKE
N004067B  (88) VASRRRMIESNLRLVVKIARRYGNRGLALLDLIEEGNLGLIRAVEKFDPERGFRFSTYATWWIRQTIERAIMNQTRTIRLPIHIVKE
N004067C  (88) VASRRRMIESNLRLVVKIARRYGNRGLALLDLIEEGNLGLIRAVEKFDPERGFRFSTYATWWIRQTIERAIMNQTVLFVCRFTS---
N004859   (88) VASRRRMIESNLRLVVKIARRYGNRGLALLDLIEEGNLGLIRAVEKFDPERGFRFSTYATWWIRQTIERAIMNQTRTIRLPIHIVKE
N004859A  (88) VASRRRMIESNLRLVVKIARRYGNRGLALLDLIEEGNLGLIRAVEKFDPERGFRFSTYATWWIRQTIERAIMNQTRTIRLPIHIVKE
N004859B  (88) VASRRRMIESNLRLVVKIARRYGNRGLALLDLIEEGNLGLIRAVEKFDPERGFRFSTYATWWIRQTIERAIMNQTRTIRLPIHIVKE
N004859C  (88) VASRRRMIESNLRLVVKIARRYGNRGLALLDLIEEGNLGLIRAVEKFDPERGFRFSTYATWWIRQTIERAL----------------
```

Section 3

```
            (175) 175  180       190       200       210       220       230       240       250      261
   EDL933 (175) LNVYLRTARELSHKLDHEPSAEEIAEQLDKPVDDVSRMLRLNERITSVDTPLGGDSEKALLDILADEKENGPEDTTQDDDMKQSIVK
  EDL933A (138) ---------------------------------------------------------------------------------------
  EDL933B  (92) ---------------------------------------------------------------------------------------
  EDL933C  (94) ---------------------------------------------------------------------------------------
    CL106 (175) LNVYLRTARELSHKLDHEPSAEEIAEQLDKPVDDVSRMLRLNERITSVDTPLGGDSEKALLDILADEKENGPEDTTQDDDMKQSIVK
   CL106A  (71) ---------------------------------------------------------------------------------------
   CL106B (153) ---------------------------------------------------------------------------------------
   CL106C  (30) ---------------------------------------------------------------------------------------
   EC3377 (175) LNVYLRTARELSHKLDHEPSAEEIAEQLDKPVDDVSRMLRLNERITSVDTPLGGDSEKALLDILADEKENGPEDTTQDDDMKQSIVK
  EC3377A (139) ---------------------------------------------------------------------------------------
  EC3377B  (71) ---------------------------------------------------------------------------------------
  EC3377C  (62) ---------------------------------------------------------------------------------------
   EC2044 (175) LNVYLRTARELSHKLDHEPSAEEIAEQLDKPVDDVSRMLRLNERITSVDTPLGGDSEKALLDILADEKENGPEDTTQDDDMKQSIVK
  EC2044A  (30) ---------------------------------------------------------------------------------------
  EC2044B (175) LNVYLRTARELSHKLDHEPSAEEIAEQLDKPVDDVSRMLRLNERITSVDTPLGGDSEKALLDILADEK-------------------
  EC2044C (175) LNVYLRTARELSHKLDHEPSAEEIAEQLDKPVDDVSRMLRLNERITSVDTPLGGDSEKALLDILADEKENGPEDTTQDDDMKQSIVK
   EC6484 (175) LNVYLRTARELSHKLDHEPSAEEIAEQLDKPVDDVSRMLRLNERITSVDTPLGGDSEKALLDILADEKENGPEDTTQDDDMKQSIVK
  EC6484A (175) LNVYLRTARELSHKLDHEPSAEEIAEQLDKPVDDVSRMLRLNERITSVDTPLGGDSEKALLDILADEKENGPEDIPRKMTI------
  EC6484B (175) LNVYLRTARELSHKLDHEPSAEEIAEQLDKPVDDVSRMLRLNERITSVDTPLGGDSEKALLDILADEKENGPEDTTQDDDMKQSIVK
  EC6484C (135) ---------------------------------------------------------------------------------------
  N004067 (175) LNVYLRTARELSHKLDHEPSAEEIAEQLDKPVDDVSRMLRLNERITSVDTPLGGDSEKALLDILADEKENGPEDTTQDDDMKQSIVK
 N004067A (175) LNVYLRTARELSHKLDHEPSAEEIAEQLDKPVDDVSRMLRLNERITSVDTPLGGDSEKALLDILADEKENGP-EIPRKMTI------
 N004067B (175) LNVYLRTARELSHKLDHEPSAEEIAEQLDKPVDDVSRMLRLNERITSVDTPLGGDSEKALLDILADEKENGPEDTTQDDDMKQSIVK
 N004067C (172) ---------------------------------------------------------------------------------------
  N004859 (175) LNVYLRTARELSHKLDHEPSAEEIAEQLDKPVDDVSRMLRLNERITSVDTPLGGDSEKALLDILADEKENGPEDTTQDDDMKQSIVK
 N004859A (175) LNVYLRTARELSHKLDHEPSAEEIAEQLDKPVDDVSRMLRLNERITSVDTPLGGDSEKALLDILADEKENGPEDTTQDDDMKQSIVK
 N004859B (175) LNRLPANRT------------------------------------------------------------------------------
 N004859C (159) ---------------------------------------------------------------------------------------
```

Section 4

	(262)	262 270 280 290 300 310 320 330 340
EDL933	(262)	WLFELNAKQREVLARRFGLLGYEAATLEDVGREIGLTRERVRQIQVEGLRRLREILQTQGLNIEALFRE----------
EDL933A	(138)	---
EDL933B	(92)	---
EDL933C	(94)	---
CL106	(262)	WLFELNAKQREVLARRFGLLGYEAATLEDVGREIGLTRERVRQIQVEGLRRLREILQTQGLNIEALFRE----------
CL106A	(71)	---
CL106B	(153)	---
CL106C	(30)	---
EC3377	(262)	WLFELNAKQREVLARRFGLLGYEAATLEDVGREIGLTRERVRQIQVEGLRRLREILQTQGLNIEALFRE----------
EC3377A	(139)	---
EC3377B	(71)	---
EC3377C	(62)	---
EC2044	(262)	WLFELNAKQREVLARRFGLLGYEAATLEDVGREIGLTRERVRQIQVEGLRRLREILQTQGLNIEALFRE----------
EC2044A	(30)	---
EC2044B	(243)	---
EC2044C	(262)	WLFELNAKQREVLARRFGLLG--
EC6484	(262)	WLFELNAKQREVLARRFGLLGYEAATLEDVGREIGLTRERVRQIQVEGLRRLREILQTQGLNIEALFRE----------
EC6484A	(256)	---
EC6484B	(262)	WLFELNAKQREVLAR--
EC6484C	(135)	---
N004067	(262)	WLFELNAKQREVLARRFGLLGYEAATLEDVGREIGLTRERVRQIQVEGLRRLREILQTQGLNIEALFRE----------
N004067A	(255)	---
N004067B	(262)	WLFELNAKQREVLARRFGLLGYEAATLEDVGREIGLTRERVRQIQV---------------------------------
N004067C	(172)	---
N004859	(262)	WLFELNAKQREVLARRFGLLGYEAATLEDVGREIGLTRERVRQIQVEGLRRLREILQTQGLNIEALFRE----------
N004859A	(262)	WLFELNAKQREVLARRFGLLGYEAATRGRCRS---
N004859B	(184)	---
N004859C	(159)	---
